# DNA transposons have colonized the genome of the giant virus *Pandoravirus salinus*

**DOI:** 10.1186/s12915-015-0145-1

**Published:** 2015-06-12

**Authors:** Cheng Sun, Cédric Feschotte, Zhiqiang Wu, Rachel Lockridge Mueller

**Affiliations:** Department of Biology, Colorado State University, Campus Delivery 1878, Fort Collins, CO 80523-1878 USA; Department of Human Genetics, The University of Utah, Salt Lake City, UT 84112 USA

**Keywords:** Genome evolution, Miniature inverted-repeat transposable element (MITE), Virus

## Abstract

**Background:**

Transposable elements are mobile DNA sequences that are widely distributed in prokaryotic and eukaryotic genomes, where they represent a major force in genome evolution. However, transposable elements have rarely been documented in viruses, and their contribution to viral genome evolution remains largely unexplored. Pandoraviruses are recently described DNA viruses with genome sizes that exceed those of some prokaryotes, rivaling parasitic eukaryotes. These large genomes appear to include substantial noncoding intergenic spaces, which provide potential locations for transposable element insertions. However, no mobile genetic elements have yet been reported in pandoravirus genomes.

**Results:**

Here, we report a family of miniature inverted-repeat transposable elements (MITEs) in the *Pandoravirus salinus* genome, representing the first description of a virus populated with a canonical transposable element family that proliferated by transposition within the viral genome. The MITE family, which we name *Submariner*, includes 30 copies with all the hallmarks of MITEs: short length, terminal inverted repeats, TA target site duplication, and no coding capacity. *Submariner* elements show signs of transposition and are undetectable in the genome of *Pandoravirus dulcis*, the closest known relative *Pandoravirus salinus*. We identified a DNA transposon related to *Submariner* in the genome of *Acanthamoeba castellanii*, a species thought to host pandoraviruses, which contains remnants of coding sequence for a Tc1/*mariner* transposase. These observations suggest that the *Submariner* MITEs of *P. salinus* belong to the widespread Tc1/*mariner* superfamily and may have been mobilized by an amoebozoan host. Ten of the 30 MITEs in the *P. salinus* genome are located within coding regions of predicted genes, while others are close to genes, suggesting that these transposons may have contributed to viral genetic novelty.

**Conclusions:**

Our discovery highlights the remarkable ability of DNA transposons to colonize and shape genomes from all domains of life, as well as giant viruses. Our findings continue to blur the division between viral and cellular genomes, adhering to the emerging view that the content, dynamics, and evolution of the genomes of giant viruses do not substantially differ from those of cellular organisms.

**Electronic supplementary material:**

The online version of this article (doi:10.1186/s12915-015-0145-1) contains supplementary material, which is available to authorized users.

## Background

Transposable elements (TEs) are mobile DNA sequences that can insert into new genomic locations, often replicating themselves during the process. Two classes of TEs exist that differ in the molecular mechanism by which they transpose from one genomic location to another – Class I TEs (retrotransposons) transpose via an RNA intermediate, whereas Class II TEs (DNA transposons) transpose via a DNA intermediate [1, 2]. A TE can be autonomous or non-autonomous; transposition enzymes for autonomous TEs are transcribed and translated from the TE’s own sequence, whereas non-autonomous TEs utilize transposition enzymes encoded by other TE loci [1].

Miniature inverted-repeat transposable elements (MITEs) are non-autonomous DNA transposons of relatively short length (100–600 bp) whose transposition requires enzymes encoded by autonomous DNA transposons [3–5]. MITE sequences include terminal inverted repeats (TIRs) and are flanked by short direct repeats (often TA or TAA) called target site duplications (TSDs). MITEs are distinguished from other non-autonomous DNA elements by relatively high copy numbers and length homogeneity [4, 5]. In addition, MITEs in both prokaryotic and eukaryotic genomes are often found close to or within genes, where they may affect gene expression or contribute exonic sequence [4–10].

TEs are widely distributed among both prokaryotic and eukaryotic genomes. TE activity has played a powerful role in the evolution of these groups, providing both the raw material for genetic innovation as well as most of the DNA content in diverse lineages [11, 12]. In contrast, TEs have only rarely been documented in the genomes of viruses. In all previously reported instances, the TEs were restricted to one or two copies per viral genome, and they were interpreted as transient passengers acquired horizontally from their cellular hosts with little to no impact on viral genome evolution [13–23]. Recently, the genomes of several giant DNA viruses within the recently proposed order “Megavirales” [24] have been shown to host other types of mobile genetic elements and repetitive, putatively mobile elements including self-splicing introns, inteins, insertion sequences (ISs), provirophages, and an atypical group of integrative linear plasmids called transpovirons [17, 25–32]. However, to the best of our knowledge, no viral genome has been reported to contain a substantial number of canonical TEs (i.e. Class I or Class II TEs that transpose via typical mechanisms) that proliferated by transposition in the viral genome.

*Pandoravirus salinus* and *Pandoravirus dulcis* are related giant viruses that likely infect amoebae of the genus *Acanthamoeba* [33]. *Pandoravirus* genomes reach 2.5 Mb, a size exceeding that of some bacterial genomes and comparable to the genomes of some single-celled, parasitic eukaryotes. *Pandoravirus* genomes are predicted to encode more than 2,500 protein-coding genes, including repetitive open reading frames (ORFs) likely generated by local gene duplications [33]. Protein-coding sequences occupy approximately 80 % of pandoravirus genomes, leaving substantial noncoding intergenic space that could harbor TEs. However, no mobile genetic elements have yet been reported in pandoravirus genomes. Herein, we identify 30 elements in the *P. salinus* genome with all the hallmarks of a MITE family, providing the first documented case of a virus populated with a canonical TE family that proliferated by transposition within the viral genome. Ten of these 30 MITEs are predicted to contribute coding sequences in the *P. salinus* genome, while others are in close association with predicted genes, suggesting that TEs were actively involved in shaping the evolution of this viral genome.

## Results

### Discovery of MITEs in the *P. salinus* genome

**Fig. 1:**
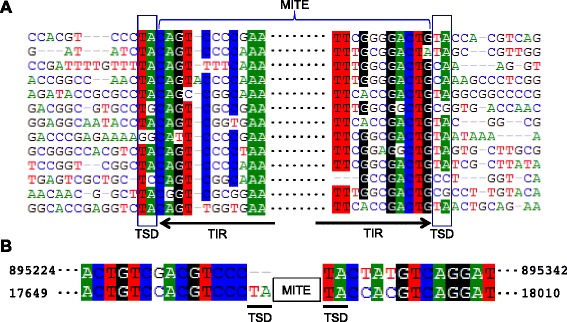
**a** ClustalW-generated multiple alignment of the 13 full-length MITEs and their flanking sequence showing partial terminal inverted repeat (TIR) sequences and target site duplication (TSD) sequences. The multiple alignment results indicate well-defined element boundaries because sequence similarity decreases dramatically outside of the MITE. **b** Pairwise alignment of the flanking sequences of a MITE insertion in *P. salinus* (bottom sequence) and a paralogous empty site elsewhere in the *P. salinus* genome (top sequence). TSD sequences (TA) are underlined. The paralogous empty site is evidence of transposition. Numbers of either side of the sequences indicate their coordinates in the *P. salinus* genome

We identified a repeat element in the *P. salinus* genome with long TIRs, a hallmark of DNA transposons. The element is present in 13 full-length copies (i.e. copies missing fewer than 5 bp of their TIRs), 13 copies >80 % of full-length, and four copies >50 % of full-length in the *P. salinus* genome. We confirmed that the repeat element has well-defined boundaries by aligning the 13 full-length copies and 60 bp of their flanking sequence (Fig. 1a). Based on an alignment of all 30 copies (Additional file 1: Figure S1), we reconstructed a 243-bp consensus sequence [Repbase ID: Submariner_Ps1] that is nearly palindromic – the TIRs are approximately 100 bp long (Additional file 2: Figure S2). Eleven of the 13 full-length copies are flanked on at least one side by a 5′-TA-3′ dinucleotide (Fig. 1a) likely representing the TSD generated upon integration into the viral genome. Four features of this element suggest that it is a MITE [3, 5]: (1) its small size (243 bp), (2) its TIRs, (3) its 5′-TA-3′ TSDs, and (4) its apparent lack of coding capacity for a transposase. Using the flanking sequences of each MITE copy as BLASTn queries against the *P. salinus* genome, we identified one paralogous site that lacked the MITE, but contained a copy of the TA dinucleotide at the insertion site (i.e. a paralogous empty site; Fig. 1b, Additional file 3: Figure S3) (sequence divergence between the flanking sequences of the MITE and the paralogous empty site is 11 % over 119 total bp; e-value = 1e-42). These data strongly suggest that the MITE spread within the *P. salinus* genome via canonical transposition events, producing 5′-TA-3′ TSDs.

### Proliferation history of the MITEs in the *P. salinus* genome

Sequence divergences of each MITE copy from the consensus sequence (i.e. the inferred ancestral sequence) range from 8–30 % (Additional file 4: Figure S4). We found no MITE copies within the genome of *P. dulcis*, the closest known relative of *P. salinus*; these two viral genomes are, on average, 65–88 % identical in orthologous coding sequence, although many of the non-coding sequences in the *P. salinus* genome do not have identifiable orthologs in the smaller *P. dulcis* genome. Together, these data indicate that the MITE may have been active since the divergence of the two pandoraviruses; however, we cannot exclude the possibility that the MITE was active in their common ancestor and subsequently lost from the *P. dulcis* genome.

### Identification of an autonomous DNA transposon related to the *P. salinus* MITEs in the genome of a potential *P. salinus* host, the amoeba *Acanthamoeba castellanii*

MITE transposition requires transposase encoded by autonomous DNA transposons. MITEs and the DNA transposons that mobilize them typically share sequence similarity in their TIRs. We looked for an autonomous DNA transposon that could have facilitated the proliferation of the *P. salinus* MITEs in both the *P. salinus* genome as well as the genomes of all species represented in public sequence databases. We found no such DNA transposon in the *P. salinus* genome using either of two methods — tBLASTn searches against the *P. salinus* genome using transposase queries representing known DNA transposon superfamilies, or the ‘Anchor’ function of the MITE Analysis Kit, which scans the genome for longer copies with putative coding sequences [34]. However, BLASTn searches using the MITE consensus sequence against the NCBI databases did retrieve one 1604 bp sequence from the genome of *A. castellanii* with sequence similarity to the MITE TIRs (coordinates AEYA01001964.1: 92260–93863). *A. castellanii* is a likely host of *P. salinus*. NCBI-BLAST2 analysis shows that this 1604 bp sequence has 29 bp TIRs, a typical feature of DNA transposons.

To determine whether the 1604 bp sequence encodes proteins associated with transposition, we queried it against the NCBI Conserved Domain Database [35]. We found that it contains a DDE superfamily endonuclease domain (e-value = 5.38e-11), suggesting that the sequence is likely a DNA transposon. We also used this 1604 bp sequence to BLASTx against the proteins encoded by TEs in Repbase to determine whether it shares sequence similarity with any known DNA transposons. We found that it encodes a protein sharing 20–29 % amino acid sequence identity with the putative transposases encoded by four Tc1/*mariner* DNA transposons described in *Acyrthosiphon pisum* (pea aphid) and *Caenorhabditis briggsae* (nematode) — Mariner-2_AP (e-value = 8e-30), Mariner-3_AP (e-value = 5e-29), Mariner-1_AP (e-value = 5e-26), and Mariner44_CB (e-value = 1e-06). These results suggest that the 1604 bp sequence is a DNA transposon of the Tc1/*mariner* superfamily.

**Fig. 2:**
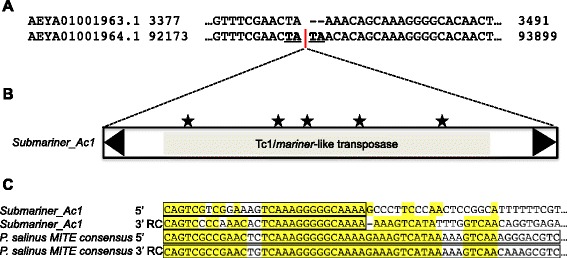
Autonomous DNA transposon in the amoeba *Acanthamoeba castellanii* that is closely related to the MITEs in *P. salinus.* **a** Pairwise alignment of the flanking sequences of the DNA transposon insertion and a paralogous empty site elsewhere in the *A. castellanii* genome. Red bar indicates the transposon insertion site. Bold and underlined letters (TA) indicate TSD. The paralogous empty site is evidence of transposition. **b** The structure of the autonomous DNA transposon in *A. castellanii*. Triangles indicate TIRs. Stars indicate stop codons in the putative transposase sequence. **c** Alignment of the ends of the consensus sequence of the MITEs in *P. salinus* and the ends of the autonomous DNA transposon sequence in *A. castellanii*, referred to as *Submariner_Ac1*. TIRs for each element are boxed. Columns in the alignment are shaded when nucleotides are conserved in at least three sequences. RC stands for reverse-complement. The sequence similarity between the TIRs of the *P. salinus* MITE and the *A. castellanii* DNA transposon *Submariner_Ac1* indicates that the *P. salinus* MITE could have been cross-mobilized in the viral genome by the *A. castellanii* DNA transposon

To investigate whether the 1604 bp sequence has been transpositionally active, we looked for paralogous empty sites within the *A. castellanii* genome. To this end, we used 100 bp of sequence flanking the 1604 bp sequence on either side as queries to BLASTn against the total genomic sequences of *A. castellanii*. We found one paralogous empty site (Fig. 2a and Additional file 5: Figure S5), confirming transposition activity of the 1604 bp sequence (sequence divergence between the empty site and its paralog containing the 1604 bp sequence is approximately 6 % over 121 bp; e-value = 1e-67). Integration of the new 1604 bp sequence generated a 5′-TA-3′ TSD (Fig. 2a), suggesting that the sequence has a typical TSD of a Tc1/*mariner* superfamily DNA transposon.

Taken together, these data show that the sequence we identified in *A. castellanii* has all the hallmarks of a Tc1/*mariner* DNA transposon. We named the sequence *Submariner_Ac1*. The transposase sequence of *Submariner_Ac1* contains many obvious disabling mutations, introducing at least five premature stop codons (Fig. 2b), strongly suggesting that it is no longer active.

Tc1/*mariner* elements are known to have produced and mobilized MITEs in many species [5, 36–39]. *Submariner_Ac1* includes TIRs that share approximately 83 % sequence identity with the TIRs of the *P. salinus* MITE consensus sequence (Fig. 2c); this level of sequence similarity is typical for autonomous DNA transposons and the MITEs they can mobilize [36, 40, 41]. Based on this sequence similarity, as well as the shared 5′-TA-3′ TSD sequences, we infer that the MITE family identified in *P. salinus* was likely derived from a *Submariner_Ac1-*like DNA transposon and subsequently amplified by a *Submariner_Ac1-*like transposase. Thus, we name the MITE family in *P. salinus Submariner_Ps1*.

tBLASTx searches using the sequence of *Submariner_Ac1* against the *A. castellanii* genome retrieved one more *Submariner*-like DNA transposon (coordinates AEYA01001733.1: 913–2735), which we name *Submariner_Ac2* (e-value = 1e-125). Like *Submariner_Ac1*, the transposase sequence of *Submariner_Ac2* also contains disabling mutations (two stop codons, one frameshift), suggesting that this element is also inactive. We find no evidence of transposition of *Submariner_Ac2* based on searches for paralogous empty sites within the *A. castellanii* genome. The sequence similarity between the TIRs of *Submariner_Ps1* (i.e. the *P. salinus* MITE family) and *Submariner_Ac2* is less than between *Submariner_Ps1* and *Submariner_Ac1*; as described above, the *Submariner_Ps1* consensus sequence retrieved only *Submariner_Ac1*, and not *Submariner_Ac2*, as a significant BLAST hit. Thus, we inferred that *Submariner_Ac2* is less likely to have mobilized the *P. salinus* MITEs, although we could not completely exclude this possibility.

### *Submariner* sequences in *A. castellanii* belong to a novel subgroup of Tc1/*mariner* DNA transposons

**Fig. 3:**
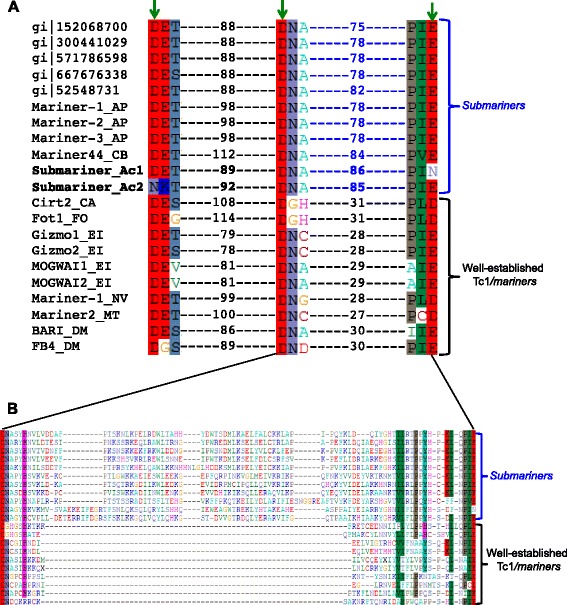
The DDE signature in *Submariner* transposases. **a** Sequences are identified by their GenBank accession numbers or Repbase IDs, if applicable. These accession numbers and Repbase IDs correspond to the nucleotide sequences from which the transposase amino acid sequences were deduced. Green arrows indicate the DDE amino acid triad that coordinates metal ion (Mg^2+^) binding during catalysis of typical cut-and-paste transposition. The DDE residues are shown within their respective conserved motifs (DET, DNA, and PIE). Numbers flanked by dashes indicate the number of amino acid positions that separate the conserved motifs based on a multiple sequence alignment. The 11 *Submariner* sequences have a much longer stretch of residues between the second D and the E residues than do representatives from the well-established Tc1/*mariner* clades. gi|152068700, gi|300441029, gi|571786598, and gi|667676338 are bacterial sequences, and gi|52548731 is an archaeal sequence; taxon information is in the text. Mariner-1-3_AP are from *Acyrthosiphon pisum* (pea aphid) and Mariner44_CB is from *Caenorhabditis briggsae* (nematode). **b** Multiple sequence alignment of the amino acids between the second D and the E residues showing the length difference between the *Submariners* and the other Tc1/*mariners*. Sequences are in the same order as in (**a**)

DNA transposons are grouped into superfamilies and smaller subclades based, in part, on shared amino acid motifs within the conserved DDE/D catalytic domain of their transposase sequence [42]. The DDE/D motif refers to the acidic amino acid triad that coordinates metal ion binding (most likely Mg^2+^) during catalysis of typical cut-and-paste transposition [43]. To determine whether *Submariner_Ac1* and *Submariner_Ac2* are part of any characterized Tc1/*mariner* subclade, we aligned (1) the putative transposase sequences from *Submariner_Ac1* and *Submariner_Ac2*, (2) the four DNA transposase hits we obtained from Repbase using *Submariner_Ac1* as a query (Mariner-1_AP, Mariner-2_AP, Mariner-3_AP, and Mariner44_CB), and (3) two representative transposases from each of five well-established Tc1/*mariner* subclades (Fot1, Pogo, Tc1, Gizmo, and Mogwai) [44]. To identify other potentially related sequences, we also performed additional BLASTx searches against the NCBI non-redundant protein sequence database using the *Submariner_Ac1* sequence as a query; we retained one representative per species from the top 20 hits (five total sequences, four bacterial and one archaeal) to be included in our alignment. Using this alignment, we examined the DDE/D signature in the *A. castellanii* sequences and found that the residues are located within conserved “DET,” “DNA,” and “PIE” motifs, indicative of Tc1/*mariner* transposases [36, 44, 45] (Fig. 3a; Additional file 6: Figure S6). The third glutamic acid residue within the conserved “PIE” motif mutated to an N in the apparently inactive *Submariner_Ac1* sequence, and the first aspartic acid residue within the conserved “DET” motif mutated to an N in the apparently inactive *Submariner_Ac2* sequence (Fig. 3a)*.* In the two *Submariner_Ac* transposases, the second aspartic acid residue and the glutamic acid were separated by a much longer stretch of amino acids (75 – 86 amino acid positions) than is found in the transposases of well-established Tc1/*mariner* clades (27 to 31 amino acid positions; Fig. 3a and b) [45]. The Tc1/*mariner* hits we obtained from Repbase (Mariner-1_AP, Mariner-2_AP, Mariner-3_AP, and Mariner44_CB) also share this long stretch of amino acids between the second aspartic acid and the glutamic acid. Finally, the archaeal and four bacterial sequences we obtained from NCBI also share this long stretch of amino acids. Based on this novel DDE signature, we inferred that the *Submariner* sequences identified in *A. castellanii* are part of a novel subgroup of Tc1/*mariner* transposons with members in all three domains of cellular life. We refer to the subgroup as *Submariner*.

We attempted to corroborate this result using phylogenetic analysis of Tc1/*mariner* transposase amino acid sequences. However, the divergence between the *Submariner* sequences and those from the five well-established Tc1/*mariner* clades, as well as the divergences among the well-established Tc1/*mariner* clades themselves, were sufficiently great that unambiguous alignment was possible for few amino acid positions (Additional file 6: Figure S6). Consequently, phylogenetic analyses of this alignment resulted in poorly supported trees (not shown).

### Submariner_Ps1 *proliferated within the* P. salinus *genome*

The paralogous empty site we identified within the *P. salinus* genome strongly suggests that *Submariner_Ps1* transposed within the viral genome (Fig. 1b). We performed additional analyses to exclude the other possibilities, namely that (1) the *P. salinus* MITEs are artifacts of DNA contamination from the *A. castellanii* genome (as *A. castellanii* was used to culture *P. salinus*), and that (2) the *P. salinus* MITEs are the result of horizontal transfer into the viral genome multiple times from another organism.

First, to test for contamination, we performed BLASTn searches using every individual copy of *Submariner_Ps1* as queries against the *A. castellanii* genome. Such searches identify only one other DNA element that shares sequence similarity with the *Submariner_Ps1* TIRs. This DNA element is a 269-bp-long MITE (coordinates AEYA01002349.1: 6514–6782; e-value = 1e-04). Because there is only one *Submariner*-like MITE in *A. castellanii*, and the sequence similarity between this *A. castellanii* MITE and *Submariner_Ps1* is restricted to their TIRs (Additional file 7: Figure S7), we can exclude the possibility that *Submariner* MITEs in the *P. salinus* genome are artifacts of DNA contamination from the *A. castellanii* genome.

Second, to test for multiple horizontal transfer events, we performed BLASTn searches using 100 bp of sequence immediately flanking all *Submariner_Ps1* insertions as queries against the *A. castellanii*, *P. dulcis*, and *P. salinus* genomes. Such searches identify three *Submariner_Ps1* insertions with flanking sequence on one side that retrieves significant hits from *P. dulcis* as well as from other locations within the *P. salinus* genome. Flanking sequence from an additional five *Submariner_Ps1* insertions retrieves hits from other locations within the *P. salinus* genome. No such BLASTn searches retrieve significant hits from *A. castellanii* (Additional file 8: Table S1)*.*

We also performed BLASTn searches against the NCBI nr database using the sequences of predicted genes present in *P. salinus* within 2 kb of *Submariner_Ps1* insertions. Such searches identify (1) 15 *Submariner_Ps1* insertions with an ortholog in *P. dulcis* on one side of the insertion, (2) two *Submariner_Ps1* insertions with orthologs in *P. dulcis* on both sides of the insertion (coordinates 196891–197139 and 2363527–2363776), and (3) one *Submariner_Ps1* insertion with an ortholog in *P. dulcis* on one side of the insertion and 100 bp flanking sequence that retrieves a significant BLASTn hit from *P. dulcis* on the other side of the insertion (coordinates 756606–756849; Additional file 8: Table S1). One *Submariner_Ps1* insertion with a *P. dulcis* predicted gene ortholog on one side is flanked on the other side by a predicted gene that retrieves significant hits from both the *P. dulcis* and *A. castellanii* genomes. No other such BLAST analyses return significant hits from the *A. castellanii* genome, although one *Submariner_Ps1* insertion is flanked by a predicted gene that retrieves a significant hit from a copepod genome (Additional file 8: Table S1). Taken together, these results show that the majority of sequences flanking the MITE insertions in *P. salinus* have homologs in *P. dulcis* and thus can be considered of ancestral viral origin prior to the spread of the MITE in *P. salinus*. These results are consistent with the idea that *Submariner_Ps1* amplified within the viral genome rather than being transferred horizontally into the viral genome multiple times from another organism.

### Genomic distribution of *Submariner_Ps1* in the *P. salinus* genome suggests exaptation

The proximity of all *Submariner_Ps1* copies to annotated *P. salinus* ORFs, detailed insertion coordinates, and the ORFs into or near which they insert are summarized in Additional file 9: Table S2. Ten out of the 30 copies of *Submariner_Ps1* are part of predicted ORFs, suggesting that these MITEs may have been exonized in the *P. salinus* genome to form novel proteins (Table 1). In eight cases, the *Submariner_Ps1* insertion extends the ORF on either the 5′ (three cases) or 3′ (five cases) end. In the other two cases, the entire predicted ORF is composed of *Submariner_Ps1* sequence (Table 1).

**Table 1:** MITEs found within annotated genes in the *P. salinus* genome

MITE coordinates in *P. salinus*	Gene associated with the MITE	Gene coordinates in *P. salinus*	Gene length (bp)	Predicted gene function	Length of overlap (bp)	MITE involved in predicted secondary structure
148208–148428	ps_155	148230–148322	93	hypothetical protein	All 93	Yes
196891–197139	ps_208	196674–196934	261	hypothetical protein	44, C end	Yes
266075–266302	ps_282	266076–266237	162	hypothetical protein	All 162	Yes
659083–659327	ps_683	658540–659259	720	hypothetical protein	177, C end	Yes
707659–707892	ps_736	707593–707739	147	hypothetical protein	80, N end	Yes
1279645–1279868	ps_1360	1276933–1279722	2790	hypothetical protein	78, N end	Yes
1298951–1299182	ps_1377	1299004–1299717	714	hypothetical protein	179, N end	Yes
2316942–2317180	ps_2397	2316744–2316953	210	hypothetical protein	12, C end	No
2363527–2363776	ps_2438	2363321–2363686	366	hypothetical protein	160, C end	Yes
2373978–2374199	ps_2448	2372753–2373989	1237	2OG-Fe(II) oxygenase superfamily	12, C end	No

Only one of the ten predicted ORFs associated with MITEs has a homolog in any other genome (Table 1). Because of the large evolutionary distance between pandoraviruses and all other known organisms and viruses, genome annotation produced a large number of predicted ORFs with no identifiable homologs in other taxa (i.e. ORFans), consistent with the results from other giant virus genome annotations [46]. In the absence of confirmatory datasets (e.g. transcriptomic or proteomic data), some predicted ORFs are likely to be false positives. To understand if the predicted MITE-associated ORFs in *P. salinus* encode amino acid sequences that form stable secondary structures, which would suggest that they may be actual protein-coding genes, we used PSIPRED [47]. All ten such translated ORFs are predicted to form some stable secondary structures (e.g. alpha helices and/or beta strands), with the MITE sequences contributing to the secondary structure in eight of the ten cases (Table 1, Additional file 10: Figure S8). In addition, because of the high coding density of the *P. salinus* genome, all *Submariner_Ps1* insertions are necessarily close to predicted ORFs, raising the possibility that they may also contribute to regulatory evolution. Although our results are suggestive, further experimental validation is required to investigate whether any MITE insertions have been exapted as new coding, or otherwise functional, sequence.

## Discussion

The discovery of giant viruses forced biologists to radically rethink previously held ideas about the upper limits of viral genome size and complexity [48]. Inspired by the early discovery of Mimivirus [25, 49], targeted searches during the past decade for new, previously undescribed giant viruses have uncovered a spectacular diversity of forms [26, 50], and the mechanisms by which they persist and reproduce within their host cells are the subject of intense research [51, 52]. Because giant viruses are so different from other viruses in genome size, particle size, and enzymatic capacity, their discovery sparked a lively debate about their origins [30, 31, 53–60]. Recent phylogenomic analyses support the independent origins of the three currently known giant virus lineages – pithovirus, the pandoraviruses, and the mimiviruses, all with genomes ≥500 kb – from ancestors within the “Megavirales” with moderately sized genomes, reflecting large-scale accumulation of sequences from multiple donors from all three domains of cellular life [17, 30, 46, 50, 61, 62]. Such genomic expansion was likely facilitated by the evolution of DNA replication machinery capable of replicating larger genomes [63].

Our results are consistent with this view of genomic expansion in giant viruses; the MITE we identify in *P. salinus* is another example of sequence accumulation underlying genome size increase. However, this particular case of sequence acquisition by a giant virus is notable for several reasons. First, to our knowledge, this is the first example of a predominantly eukaryotic canonical Class II TE (i.e. a Tc1/*mariner*) colonizing a giant virus, although other TEs (e.g. IS sequences of the bacterial and archaeal IS*607* family) have previously been reported in giant virus genomes [17, 30, 31, 46]. Second, we present evidence suggesting that the MITE in *P. salinus* transposed within the viral genome. In contrast, evidence that other TEs have transposed within viral genomes has been lacking, although previous studies reported this as a possibility [17, 30, 31]. Third, the MITE in *P. salinus* is present at high copy numbers relative to TEs in other viral genomes and, based on predicted ORFs, some of these copies may have contributed novel protein-coding sequence to the virus.

More generally, comparative genomic analyses across the three domains of cellular life and numerous viral lineages are revealing a complex picture of horizontal transfers among genomes; transfer rates differ among donor/recipient pairs as well as among types of sequences [64–66]. Given such asymmetries, mobile genetic elements that have overcome impediments to colonization across the multiple domains, as well as the viruses, are important models for understanding what limits horizontal transfer across, and outside of, the Tree of Life. IS*607* sequences are one such mobile element. These primarily prokaryotic sequences have colonized some eukaryotes as well as giant viruses, although their capacity for transposition outside of prokaryotes remains uncertain [66]. Herein, we demonstrate that Tc1/*mariner* TEs are another such mobile genetic element. Previously, Tc1/*mariner* elements and their MITE derivatives had been identified in a wide variety of protozoans [42, 67, 68], plants [69], fungi [37, 70], and metazoans [71, 72], and their related prokaryotic IS sequences had been identified in diverse bacteria and archaea [73, 74]. We report the colonization of a giant virus genome by a MITE derived from an apparently novel Tc1/*mariner* subgroup with representatives from all three domains of cellular life, expanding the range of this superfamily of TEs even further.

How might the *Submariner_Ps1* MITEs have colonized and spread within the *P. salinus* genome? Based on TIR sequence similarity, as well as the fact that *Acanthamoeba* is a likely host of *P. salinus*, it is quite possible that the amoeba-encoded *Submariner_Ac1* transposase once mobilized *Submariner_Ps1* MITEs in the *P. salinus* genome. However, the viral MITEs are unlikely to have originated as an internal deletion derivative of *Submariner_Ac1* because sequence similarity between the two transposons is largely restricted to their TIRs (Fig. 2c). Thus, *Submariner_Ps1* MITEs likely trace their origin to an autonomous transposon related to, but distinct from, *Submariner_Ac1*. This progenitor element could have occurred in the viral genome or the genome of the viral host (i.e. *A. castellanii* or another *Acanthamoeba*)*.* Alternatively, because free-living amoebas ingest a variety of microorganisms through phagocytosis, many of which are resistant to digestion and stably coexist “in sympatry” within the amoeba [75, 76], the progenitor element could have occurred in another amoebal symbiont. Extensive horizontal transfer of sequences among prokaryotic, eukaryotic, and viral microorganisms that stably coexist inside amoebas, as well as the host amoeba itself, has been reported, demonstrating that free-living amoebas serve as “melting pots” for genome evolution [17, 50, 66, 75–77]. Irrespective of the original source of the *Submariner_Ps1* MITEs in *P. salinus*, we show a new combination of ingredients within this “melting pot” — a canonical TE within the genome of a giant virus.

## Conclusion

Pandoraviruses were named in reference to the surprises their unusually large genomes likely concealed [26]. Herein, we have shown that the *P. salinus* genome has been colonized by a MITE derived from the Tc1/*mariner* superfamily of Class II DNA transposons, and that this MITE was likely mobilized within the viral genome. We have shown that an autonomous Tc1/*mariner* DNA transposon related to this MITE is present in the genome of a likely pandoravirus host, the amoeba *A. castellanii.* Our discovery highlights the remarkable ability of DNA transposons to colonize and shape genomes both across, and outside of, the Tree of Life. Our findings continue to blur the division between viral and cellular genomes, adhering to the emerging view that, despite fundamental differences between cellular organisms and viruses (e.g. reproduction by cell division versus virion production) [54], the content, dynamics, and evolution of the genomes of these different biological entities do not substantially differ from one another [78–81].

## Materials and methods

### Dataset

We downloaded genomic sequences of two pandoraviruses from GenBank [82] (*P. salinus* and *P. dulcis*; accession numbers KC977471 and KC977470, respectively). We also downloaded the assembled contigs (assembly version Acas_2.0) for the free-living amoeba *A. castellanii* (accessions AEYA01000001 to AEYA01002545) from GenBank.

### Identification and characterization of repetitive sequences in pandoravirus genomes

We used RepeatScout (version 1.0.5) [83] to identify *de novo* repeats from the genomic sequences of *P. salinus* and *P. dulcis*; the l-mer length was set to 15 and other parameters were set to default values. Only repeats that were >50 bp in length and <50 % low-complexity sequence were included in downstream analysis. We used RepeatMasker (version 3.2.9, [84]) to identify the overall repeat content of each genome based on the corresponding custom repeat library generated with RepeatScout. The search engine for RepeatMasker was Cross_Match [85]. To confirm the boundaries of the repeat element identified in the *P. salinus* genome, we extracted the sequences of all full-length copies (minus ≤5 bp at each end), along with 60 bp of flanking sequences. We performed multiple sequence alignment of the 13 full-length elements, along with the 60 bp of flanking sequence, using ClustalW implemented in BioEdit (version 7.2.0) [86], and the alignment results were visualized in BioEdit, shading identities and similarities (shade threshold 75 %). We predicted the secondary structure of the repeat element using the mFold web server [87, 88].

### Identification of a possible autonomous partner for the MITEs in the *P. salinus* genome

We looked for an autonomous DNA transposon that could have facilitated the proliferation of *P. salinus* MITEs in both the *P. salinus* genome as well as the genomes of all species with representation in public sequence databases*.* We used two independent methods to search the *P. salinus* genome. First, we used all the known proteins encoded by DNA transposons as queries to tBLASTn against the DNA sequences of the *P. salinus* genome, with an e-value cutoff of 1e-5. We excluded helicase, encoded by rolling circle DNA transposons (i.e. Helitrons), because they are not known to generate MITEs. Second, we used the consensus sequence of the *P. salinus* MITEs as the input for the Anchor function of the MITE Analysis Kit [34, 89] to retrieve longer elements bearing similar terminal sequences and coding sequences whose products share sequence similarity with known proteins encoded by DNA transposons. We checked the output of the MITE Analysis Kit manually to remove false output entries. We obtained the protein sequences encoded by DNA transposons used in these two methods from the TE-encoded protein database, available in the downloaded RepeatMasker package [90].

Next, to search for possible autonomous partners of the *P. salinus* MITEs in other genomes, we used the consensus sequence of the *P. salinus* MITEs as the query for homology searches (BLASTn) against the NCBI databases (Nucleotide collection, EST, STS, GSS, WGS, TSA, HTGS, last accessed on 2014 June 1). Finally, to identify other *P. salinus* MITE-related sequences in the *A. castellanii* genome, we used every MITE sequence identified in *P. salinus* as queries for homology searches (BLASTn) against the locally installed most recent assembly of the *A. castellanii* genome (Acas_2.0), and we manually checked every obtained hit.

### Characterization of the possible autonomous partner for the MITEs in the *P. salinus* genome

We found a 1604 bp sequence representing a possible autonomous partner of the *P. salinus* MITEs in the *A. castellanii* genome. To characterize this putative transposon, we (1) used NCBI-BLAST2 to identify its TIR, (2) queried it against the NCBI Conserved Domain Database [35], and (3) queried it against the TE-encoded protein database [90] using BLASTx (e-value ≤1e−5). To identify potential paralogous empty sites, we used 100 bp of its flanking sequences as queries to BLASTn against the genomic sequences of *A. castellanii*. Based on our results, we named the putative transposon *Submariner_Ac1*, and we named the related MITE in the *P. salinus* genome *Submariner_Ps1.* To look for other related sequences within the *A. castellanii* genome, we used BLASTn with the *Submariner_Ac1* sequence as the query.

To determine whether *Submariner_Ac1* and the related *Submariner_Ac2* belong to any well-characterized clade of Tc1/*mariner* DNA transposons, or to a previously uncharacterized clade, we used the complete nucleotide sequence of *Submariner_Ac1* for homology searches (BLASTx) against the NCBI non-redundant protein database (nr). We examined the top 20 hits and kept one representative from each species not already represented in our BLASTx results from Repbase; this yielded five total sequences (four bacterial sequences – *Beggiatoa* sp. PS, gi|152068700; Deltaproteobacterium NaphS2, gi|300441029; *Candidatus* Magnetoglobus multicellularis str. Araruama, gi|571786598; and *Desulfobacula* sp. TS, gi|667676338; and one uncultured archaeal sequence, GZfos18F2, gi|52548731). These accession numbers correspond to the nucleotide sequences from which the transposase amino acid sequences were deduced. We aligned these five sequences, *Submariner_Ac1* and *Submariner_Ac2*, the four hits we retrieved from Repbase, and two sequences from each of the five well-characterized clades of Tc1/*mariner* DNA transposons (Repbase IDs: Cirt2_CA, Fot1_FO, Gizmo1_EI, Gizmo2_EI, MOGWAI1_EI, MOGWAI2_EI, Mariner-1_NV, Mariner2_MT, BARI_DM, and FB4_DM) using PSI-Coffee, an aligner within the T-Coffee multiple alignment package that aligns distantly related protein sequences using homology extension [91, 92]. Based on this alignment, we identified the DDE catalytic amino acid triad, their associated conserved motifs, and their intervening sequences of amino acids.

We then generated a similar alignment, but including a non-Tc1/*mariner* transposase (Merlin1_CB) as an outgroup. We retained only amino acid positions with alignment scores of “good” (143 amino acid positions) and performed Bayesian phylogenetic analysis using a mixed model of amino acid substitution, implemented in MrBayes 3.2 [93]. We ran the analysis for 10,000,000 generations, sampling every 1000, with three heated chains. Twenty-five percent of the sampled trees were discarded as burn-in and convergence was verified by comparison of the average deviation of split frequencies between two independent runs. The limited phylogenetic signal in this short alignment yielded an unresolved tree. We limited the scope of our analysis to sequences within the Submariner subgroup (*Submariner_Ac1*, *Submariner_Ac2*, Mariner-1_AP, Mariner-2_AP, Mariner-3_AP, and Mariner44_CB, and the four bacterial sequences – gi|152068700, gi|300441029, gi|571786598, gi|667676338 – and one archaeal sequence – gi|52548731 – we identified from Genbank) and an outgroup from one of the well-characterized Tc1/*mariner* clades, performing alignment and phylogenetic analysis as above. In all cases, the distance to the outgroup resulted in low numbers of unambiguously alignable amino acid positions and spurious root placement, demonstrated by the different root attachment points recovered depending on the outgroup sequence used.

### Examination of *Submariner_Ps1* proliferation dynamics in the *P. salinus* genome

To characterize the proliferation history of *Submariner_Ps1* in the *P. salinus* genome, we calculated the sequence divergences of *Submariner_Ps1* elements from their consensus sequence using RepeatMasker, binned the divergence values, and plotted them as a frequency histogram. To determine whether *Submariner_Ps1* proliferation occurred within the *P. salinus* genome, or whether the multiple *Submariner_Ps1* insertions resulted from independent introductions from a different genome (e.g. a viral host), we used 100 bp of immediately flanking sequence from all 30 *Submariner_Ps1* insertions to query both amoeba and pandoravirus genomes using BLASTn. We also used the sequences of all of the predicted genes present in *P. salinus* within 2 kb of each *Submariner_Ps1* insertion as queries to BLASTn against the NCBI nr database.

### Examination of *Submariner_Ps1* exaptation in the *P. salinus* genome

We summarized the locations of all *Submariner_Ps1* copies relative to annotated *P. salinus* genes to assess whether *Submariner_Ps1* insertions were within, or in close proximity to, predicted ORFs. We used PSIPRED [47] to predict the secondary structure of the translated MITE-associated ORFs [94].

## Additional files

Additional file 1: Figure S1. Multiple sequence alignment of all 30 miniature inverted-repeat transposable element (MITE) copies. Sequences are named by their coordinates in the *P. salinus* genome. Additional file 2: Figure S2. The secondary structure of the *P. salinus* miniature inverted-repeat transposable element (MITE) consensus sequence [Repbase ID: Submariner_Ps1] predicted by mFold. Additional file 3: Figure S3. Pairwise alignment of a site with a miniature inverted-repeat transposable element (MITE) insertion (bottom sequence) and its paralogous empty site (top sequence) in *P. salinus*. Target site duplications are underlined in red. Additional file 4: Figure S4. Frequency histogram showing pairwise divergences between individual MITE insertions in *P. salinus* and the miniature inverted-repeat transposable element (MITE) consensus sequence. Additional file 5: Figure S5. Pairwise alignment of a site with a DNA transposon insertion (bottom sequence) and its paralogous empty site (top sequence) in *A. castellanii*. Target site duplications are underlined in red. Additional file 6: Figure S6. Multiple sequence alignment of (1) the putative transposase sequences from *Submariner_Ac1* and *Submariner_Ac2*, (2) the four DNA transposase hits we obtained from Repbase using *Submariner_Ac1* as a query (Mariner-1_AP, Mariner-2_AP, Mariner-3_AP, and Mariner44_CB), (3) two representative transposases from each of five well-established Tc1/*mariner* clades (Fot1, Pogo, Tc1, Gizmo, and Mogwai), and (4) the five hits obtained from the NCBI non-redundant protein database (nr) using *Submariner_Ac1* as a query (four bacterial sequences — *Beggiatoa* sp. PS, gi|152068700; Deltaproteobacterium NaphS2, gi|300441029; *Candidatus* Magnetoglobus multicellularis str. Araruama, gi|571786598; and *Desulfobacula* sp. TS, gi|667676338, and one uncultured archaeal sequence, GZfos18F2, gi|52548731). Sequences are identified by their GenBank accession numbers or Repbase IDs, if applicable, which correspond to the nucleotide sequences from which the transposase amino acid sequences were deduced. The multiple alignments were generated by PSI-Coffee, an aligner within the T-Coffee multiple alignment package that aligns distantly related protein sequences using homology extension [83, 84]. Red arrows indicate the DDE amino acid triad that coordinates metal ion (Mg^2+^) binding during catalysis of typical cut-and-paste transposition. Additional file 7: Figure S7. Pairwise alignment of the miniature inverted-repeat transposable element (MITE) in the *A. castellanii* genome and the MITE in *P. salinus* (*Submariner_Ps1*) that has the highest sequence similarity (out of the 30 MITE copies) with the MITE in *A. castellanii*. Additional file 8: Table S1. The search for homologs of the *P. salinus* miniature inverted-repeat transposable element (MITE) flanking sequences in other genomes. Additional file 9: Table S2. Proximity of *P. salinus* miniature inverted-repeat transposable elements (MITEs) to annotated *P. salinus* genes. Additional file 10: Figure S8. Predicted secondary structure of the hypothetical protein encoded by *P. salinus* gene Ps_1377, one of the predicted *P. salinus* open reading frames that includes miniature inverted-repeat transposable element (MITE) sequence. In this example, the first 60 amino acids of the predicted protein are MITE-derived and form three stable secondary structures: one β-strand and two α-helices. Secondary structure prediction was done using PSIPRED.

## Abbreviations

IS: Insertion sequence
MITE: Miniature inverted-repeat transposable element
ORF: Open reading frame
TE: Transposable element
TIR: Terminal inverted repeat
TSD: Target site duplication
