# Supplementary material for: DNA transposons have colonized the genome of the giant virus Pandoravirus salinus
Source: BMC Biol. 2015 Jun 12;13:38. doi: 10.1186/s12915-015-0145-1 (PMC4495683; doi:10.1186/s12915-015-0145-1)
Supplement: Additional file 1: Figure S1. — Multiple sequence alignment of all 30 miniature inverted-repeat transposable element (MITE) copies. Sequences are named by their coordinates in the P. salinus genome. [file 12915_2015_145_MOESM1_ESM.pdf]

```
1627555-1627762   1 ..........CAAACTGTTG...CGAGGGGTAACCAAAAACCGAGTCCC....TAAAAGGAC.........CAACAAAAACAC.ACCA
1298951-1299182   1 .................TCA......AAAGGAACAGAAG.AA.AGTCGT.......AAAGGC............ATGTGTCGG.GTCA
2317326-2317510   1 TACAGT.CGGTGAACTCACA......AAAGGGGCGAAA..AA.AGTCAT.....AATTGGTC...........AACTTTGCGT.....
266075-266302     1 ............................................GTCAC.....AAAAAGTC...........AACAGAGCAT.CCCA
1336186-1336424   1 ..........CAAATTATCA......AAGGAGGCCGGAA...............GGAAGGTC...........AACGTATCGC.CTCA
2261535-2261757   1 ...............................GGCAAAGG.AA.AGTCAC.....AAAAAGTT.............GACAGCGT.CCCA
2373978-2374199   1 .............ACTCTTG......AATAGACCAAAAC.AA.AGTCGTCAAAAAAAGGGTC..........AACAAAAGCGCACCAA
1279645-1279868   1 ...........GAACCGCCA......AAGGCAGCAAAAGAAAAAGCCAT..CGGCGAAAGCC...........AAAGGGACAT.CCCA
2363527-2363776   1 TACGGT.CGCGGAACTGCCA......AAGGAGGCGAAAG.AA.GACGAC.....AAAAGATCG........TCGACAAAGCATCCCAA
659348-659617     1 TGCAGT.CGCCGAACCGCCAAGAGGGGGAGGGACGAGAC.AA.GGCCAT.AAAAAGATAGTC...........GGAGGGATGT.CGCA
1401197-1401418   1 ............................GAGGGCAAAAT.AA.GGCCAT.....GAAAGGCC...........AACAAAGCGT.TCCA
10194-10372       1 TACAGT.CCCCAAACTCTCGAATGGGGGGGGGGCAAAAT.AT.AGTCAC.....AAAAAGTC...........AAAGGGGCGC.TCCA
671107-671355     1 ..CATT.CCCCGAATTACCA......AAGGGGGCAAGAG.GG.GACCAT..AAAAAAAAGTT...........AACAAAGCGT.CCCA
707659-707892     1 .......CGCCAAACTGTCA.....AAGGGAGGCAAAAG.AA.AGTCAC..AAAAAAAAGTC...........GACAAAGCGT.CTCG
1782915-1783142   1 ..CAGT.CGGCAAACTGTCA......AAGGGGGCAAAAC.AA.AGTCAT....AAAAAAGCC...........AACAAAGCGT.CTCA
148208-148428     1 .................TCA.....AAGGAGGGCAAAAG.AAGGGTCAC.....GAAAAATC...........AAAGGGACGC.CCCA
527083-527339     1 TACAGTACCCCAAAGCGTCA....AAGGGGCGGCAAAAG.AA.AATCAT....AAAAAAGTC.............GAGGACGCCCCCA
2320023-2320259   1 .....T.CTCCCAAATCTCA......AAGAGGGCAAAAG.AA.AGTCATAGGAAAAAAAGTC...........GGAGCGGCGT.CCCA
399024-399164     1 TGCAGT.CCGCAAACTGTCC......AAGGAGCCAAAAGAAA............AAAAAGTCGCAAGCCAACAAAAAGAGTGT.CCCA
196891-197139     1 TGCGGT.CTCTGGACCGCCA.....AAGAGGGGCAAAAG.AA.AGTCAT...TAAAAAAGTG...........AAAGGAATGTCCCCA
659083-659327     1 TACAGC.CGGCAAACTTCCA......AAGGGGGCAAAA..AA.AGTCAT.....AAAAAGTC...........AACAAAGCGT.CCCA
756606-756849     1 TACAGT.CCCCTAACTGTCA......AAGGGGGCAAAAC.AA.AGTCAT.....AAAAAGTC...........AGGGGGGCGT.CCCG
1436862-1437108   1 TACAGT.CCCCAAACTGCCA......AAGGGGGCAAAAC.AA.AGTCAT.....AAAAAGTC...........AGACGCACGT.CCCA
349347-349598     1 TACAGT.TTTCAAATTGCCA.....AATGGCAACAAAAG.AA.AGTCAT....AAAAAAGTC...........AGAGCGACGT.CCCA
1732017-1732183   1 ....................................................................................CCCA
2316942-2317180   1 TACAGC.CGGTGAACTCTAA.....AAGGGGGGCAAAAC.AA.AGTCAT.....AAAAAGTC............AAAGGATGT.CTTA
662519-662768     1 TACAGT.CGGTGAACTCTCA......AAGGGGCCAAAAC.AA.AGTCGT.....AAAAAGTC...........AAATAGATGT.CCCA
2366989-2367235   1 TACAGT.TGGTGAACTCTCA......AAGGGGGCAAAAC.AA.AGTCAT.....AAAAAGTC...........GAATAGATGT.CCCA
29444-29711       1 TACAGT.CCCCAAACTCTCATTGGGGGGGGGGGGGGAAT.AA.AGTCAT...AAAAAAAAGCC...........AAAGGGACGT.CCCA
17707-17952       1 TACAGT.CCCCGAACCCTCA......AAGGGGGCAAAAT.AA.AGTCAT.....AAAAAGTC...........AAAGGGACGT.CCCA
```

```
1627555-1627762   66 GGTATCGAGTGT.CTTGT.T......TGGACTGCTGAG.GGGGAATGCGCCGACGCGA........CGGCATC..CCAT...CTTTCT
1298951-1299182   48 CGCCTAATGTCT.GTTGT.T......TCATATGTGTGGAACAAAAAGGGTATTGGCGC.................ACACTTGTGCGCG
2317326-2317510   60 CGTGCAGATCTT.TTCACACGCAAAACTGTCTGCTTG.........GCGTCGACAAGAAAAATTTGTG....GGGACCT...GTCT.G
266075-266302     34 CGCATACAGGCC.ATTGT.T......TTGCCTCGTTG..AAA..AATGGCAAACAGAATGG.....GCGCAAGAGACAT.CTATCTGA
1336186-1336424   52 TGCCTACAGCCT.GTCGG.T......TTGATTGCCT...AGA..AAGTGC................CGACAGCAGGCAT...GCCTTT
2261535-2261757   41 TTTTTGCGGCCT.GTCGC.T......GTGCCTAGGTG..GGA..ATGCCCAAAAGTCC........CAACATTGGATAT...GTCTCA
2373978-2374199   60 .GTCTGCAGCCCAGTTGT.T......CTGCCTGCTTG..AGCCTGAGCG.GAAAGCGT........CGACAATAAACATGGCTTGTTG
1279645-1279868   65 TGTCTGTGGCGC.ATTGT.T......TTATCTTTTTA..ACA..ATTTGCAGACACTT........GGCGGTGAGGCGT...GCCCTG
2363527-2363776   71 CGCCTACAGCCC.GCCCT.T......GTGCCTGCTTG..GGA..ATGCGCCAAAGCGC........CGACATCGGACAC...GCAAAG
659348-659617     77 TGCACGCAGCCC.GCTGCCC......TTGTCTGCTC...AAA..AATGGCAGGCATGCCGGGGGGGGGGGCGAGACAT...GTCTGA
1401197-1401418   46 TGTCTACAGTCT.GCTAT.T......TCCCCCGCTTG..GGA..ATGTGTCAGAGTGC........TGGCACCAAACAT...ATTTCA
10194-10372       73 TGTCTGTGGCCT.GCTGT.T......TTGCCTTCTCG..GGC..ACATGCAAAAGCGT........TGACAACGGACAT...GTCTCA
671107-671355     68 TGTCTTCGACCC.ACTGT.T......TTGTCCGCTT...AAA..AATTGTAGACATAT........GAGGGCGGGGCAT...GTCTGG
707659-707892     65 TGTCTGCGGCCC.GTCATTT......TTGGCTGCTTG..GGA..AGGTGCCAGAGCGC........CAACGCGAGACAT...GTCTTG
1782915-1783142   66 TGTCTACAGCCC.GTTGT.T......TTGCCTGCTTA..AAA..AATTATAGACAAAA........TGGATAGGGACAT...GTCTGG
148208-148428     53 TGTGTGCAGTCT.GTTGT.C......TTGCCTGCTCGAAAAAAAAAGCACGGGCATCT........GGTGGTGGGTCAT...GTCTGG
527083-527339     70 TGCCCGCGGCCC.GTTGT.T......TTATCTGCTTA..AAAGGACATGTGGGCATGT........GGGGCCGAGGCAT...GTCTGG
2320023-2320259   67 TGCGCGCGGTCC.GTTGC.T......CTGTCTGCTTG..A.A..AATTGTGGACAAGC........G..CGCGAGACAT...GCCTAA
399024-399164     73 TGTCTACAGCCC.GTTGT.G......TTGCCGGCCCGA.AAA..ATGCGCAAAGGTGC........CGACATTGGCCAT...GTCCCG
196891-197139     71 TGTCCCCAGCCC.GTTGT.T......TTGTCTGCTTG..A.A..AATGGCGGACGCAC........GGGTGCGGCGCAT...GCCTGG
659083-659327     66 TGTCTACGGCCT.GCTAT.T......TCGCCTGCTTG..GCG..ATGCGCCAAAGTGC........CGACGCCAGGCAC...GCCTCG
756606-756849     67 TGTCTACAGCCT.ATTGT.T......TTGCCCGCTTG..GGA..ATGCGCCAAAGTGC........CGACACCAGACAT...G..TCG
1436862-1437108   67 TGTCTACAGCCT.GTTGT.T......TTGCCCGCTTG..GGA..ATGCGCCAAAGTGC........CGACACCGGACAT...GTCTCA
349347-349598     69 TGTCTACAGCCT.GTTGTCT......TTGTCTGCCTA..C.A..AATTCTAGACAAAT........GGAGATGAGACAT...GTCTGC
1732017-1732183    9 TGTCCGCAGCCC.GTTGT.T......TTGTCTGCTTG...AA..AAGTGTAGACAAAT........GGATACGAGACAT...GTCTGA
2316942-2317180   67 TATCTACAGCCT.GTTGT........TTGCCTGTTCG..ATA..AACTGTCGACAAAT........GAAGATGAGACAT...ATATGG
662519-662768     67 TGTCTACAGCCT.GTCGT.T......TTGCCCGCTTG...AA..AATTGTAGACAAAT........GGACATGGGACATGTCGTCTGC
2366989-2367235   67 TGTCTACAGCCT.GTTGT.T......TTGCCCGCTTG...AA..AATTGTGGACAAAT........GGACATGGGACAT...GTCTGC
29444-29711       75 TGTTTGCAGCCC.GTTGT.T......TTGTCTGCTTA..A.A..AATTGTAGACGTAC........GGGGGCGAGATAT...GTCTGA
17707-17952       67 TGTCTACAGCCT.GTTGT.T......TCGTCTGCTTA..A.A..AATTGTAGGCATAT........GGGGGTGAGACAT...GTCTGA
```

```
1627555-1627762   135 ...CGCCCGCGCGCGCGCGCGTCTGC...TGAAATTCTGTCGGCAGACG...ACACAACAGATTGTAGGCATTT.TTT.GGGGCG...
1298951-1299182   114 ........GCGTGGTGCGCCAATTTG...CGA..........TCTGACA...AAGGAACAGACTGCAGACACTTTTTCGAGGCGATG
2317326-2317510   133 ...CGGCAGTTGCGC..CAGACCGCC...CAA..........GCAGGCA...AATTTGCGGTGTGTAGACACTT..............
266075-266302     107 ...CGGC.ACTTTGG..GGCGTCTCCACACAC..........ACAGGCG...GAACAATGGCATGTAGGCATCCATTT.GGATTGCTC
1336186-1336424   115 ATGCGTCTACTTCTG..CAAA.TTTT...GAA..........GCGGACAAA.AAACAGCAGGCTGTTGGCAG.T.TTT.GTGACCCTT
2261535-2261757   109 ........ACCATAGGTCACGGTTGT...CGA..........GCAGACA...AAACAACGGACTGCAGACGCTT.TTT.GGGACGCTC
2373978-2374199   132 ...ATAC.ACCCTCA..ATTT.TTTT...CGA..........GCAGGTA...AAACAACGGGACGCAAACAC.T.TTC.AGGACGTCG
1279645-1279868   133 ...GGGC.GCTTTTG..TGCA.TTCC...CGA..........CCAGGCA...AAACAATAGGCCGCAGACAT.T.TTT.TGGGCACTG
2363527-2363776   139 ...CGAC.ATGTCTA..CATT.TTTT...TGA..........GCAGATG...AAACAAAAGGCCGTAGACAC.T.TTT.GGGACGTCC
659348-659617     153 ...CGAC.ATTTTGG..GGCA........CCG..........ACAGGCAA..AACCAACAGGCTGTAGACGT.T.TTT.AGGATGCTT
1401197-1401418   114 ........GCATTTG....CATTGTT...TAG..........GCAGACA...AGACAACAGGCTGTAGATAGTT.TTT.GCGATGTCG
10194-10372       141 ...C..TTATGCGTC..TGCAATTTT...TGA..........ACAGGCA...AAACAACGGG..........................
671107-671355     135 ...TGGC.GCTCCCG..CGCA.TTCA...CAA..........GCAGGCA...AAACAACAGGCCGTAGGCGC.T.TTT.GGGACAGTT
707659-707892     134 ...CCCCTATATTTA..CATTTTTTT...CGA..........GCAGGCAA..AAACAGCAGACCCTAGACACAT.TTG.GGGACATCC
1782915-1783142   134 ...CGGT.ACTTTGA..CGCA.TCGC...CAA..........GGAGGGC...AAATAGCAGGTTGTGGGTGC.T.TTC.GGAACATCC
148208-148428     125 ...CGGC.ACTTTTG..GCCA.TTCC...CAA..........GCAGGCAA..AAAGGACAGGCCGCAGGCGC.T.TTT.GGGACGCGC
527083-527339     140 ...CGCC.GCTTTCG..CGCA.TTCC...CAG..........GCAGGCA...AAACAACAGGCTGCAGATAC.T.TTT.AGGACGCTT
2320023-2320259   132 ...CAGT.GCCTTTG..TGCC.CTCG...CAA..........GCAGGGA...AACCAACAGGCTGCAGACAC.C.TTT.GGGACGCCG
399024-399164         ........................................................................................
196891-197139     138 ...CGGC.ACTTTTG..CGCA.TTCC...CAA..........GCAGGAAAAAAAACAACAGCCTGTTGGCAC.T.TTT.GGGACGCTT
659083-659327     134 ...C..C.ACCTGTC..TACATTTTT...CAA..........GCAGGCA...AAACAACGGGATGTAGACAC.T.TTT.GGGACATCC
756606-756849     133 ...C..C.ATTTGTC..TACAATTTT...CAA..........GCAGGC....AAACAACAGTCTGTAGACAC.T.TTT.GGGACGTCC
1436862-1437108   135 ...C..C.ATTTGTC..TACAATTTC...TAA..........GCGGGCA...AAACAACGGTCTGTAGACAC.T.TTT.GGGACGGTT
349347-349598     137 ...TGGC.ACTTTGG..CGCA.TTCC...CAA..........GTAGGCA...AAAGAGCAGGCCGTAGACAC.T.TTT.GGGACGCTT
1732017-1732183    76 ...AGGC.ACTTTCG..CGCA.TGCC...TAA..........GCAGGCA...AAACAACTGGCTGCGGACGC...TTTCAGGACGCTT
2316942-2317180   134 ...CGGC.ACTTTGG..CGCA.TCCC...CAA..........GCAGGTG...AAATAACGGGCTGTAGACAC...TTCTGGGACACCT
662519-662768     137 ...CGGC.GCTTTAG..CGCA.TTCC...CAA..........GCAGGCG...AAATAGCAGGCTGTAGACAC.T.TTT.GGGACGCTT
2366989-2367235   134 ...CGGC.GCTTTGG..CGCA.TTCC...CAA..........GCAGGCG...AAATAGCAGGCTGTAGACAC.T.TTT.GGGACGATT
29444-29711       142 ...CGGC.ACTTTCG..CGCA.TTCC...CAA..........GCAGGCA...AAACAACGGGCTGCAAACAC.T.TTT.GGGACGTCC
17707-17952       134 ...CGGC.ACTTTCG..CGCA.CTCC...CAA..........GCGGACA...AAACAACAGGCTGTAGACA..T.TTT.GGGACGCCC
```

```
1627555-1627762         ..........................................................
1298951-1299182    185  CTTTT....GTTGTTTTCTTTTA..TTCCTTTTTTTTTTCGAGAGTCTGGGGAC....
2317326-2317510         ..........................................................
266075-266302      182  TCCTTTT..GTGGCCTTCTTATC.........CACCCGCTGGCAGTCTGGGGACCGTA
1336186-1336424    190  TCTTCTTGCGACCTTTTGTTTAG........CCCCCATTTGAGAGTTTGGCAAGTGCA
2261535-2261757    178  CTTTTT...GTGGATTTATCTCA.........CCCCCTTTAACAGTTTAGACGATCGT
2373978-2374199    201  TGTTTAGGACAGTTTTTATTTT....................................
1279645-1279868    202  TGTTT.....AGCATTTCCTTTG.........CCCTC.....................
2363527-2363776    208  TCGCA....ATGATTTTCTTTTG.........TCCCCCCTGACAGTTTGGCGACTG..
659348-659617      219  TTTTTTTT.ATGATCTTCTCTTG...CCTCCCCCCTCCTTGGCAGTTTGGCGGCTG..
1401197-1401418    179  TTTTT....ATCGCCTCCTTTTG..........CCCCTTTGACAGTTTGGGGACTTTA
10194-10372             ..........................................................
671107-671355      204  TTATTT...ACGACTTTCTTTTG.........TCCCCTTTGACAATTCGGCGACTGTA
707659-707892      207  TTTTT....ATGATTTTCTTAGG.........TCCCTTTCG.................
1782915-1783142    198  .........ATGACTTTGTTTTG.........CTCCCTTTGGCCGACTG.........
148208-148428      195  TTTTT....ATGACTTTCTTTTG.........TCCTCTTT..................
527083-527339      209  TTTTTTACAACAACTTTCTTTTA.........CTCCCCCCAACAGTTTGGGGACTGCA
2320023-2320259    201  TTTTTTCT.ATGACTTTCTTTTG.........CCCTCTTTGAAAGTT...........
399024-399164           ..........................................................
196891-197139      210  TTCTTTTT.GTGACCTCCTTTTG........CCCCCTTTGGGCAGTTTG.........
659083-659327      202  TTTTT....ATGACTTT.TTTTG.........CCCCCTTTGAGGGTTCACCGACTGTA
756606-756849      200  TTTTT....ATGACTTTGTTTTG.........CCCCCTTTGATAGTTCGGAGGCTGTA
1436862-1437108    203  TTTTT....ATGACTTTGTTTTG.........CCCCCTTTGACAGTTCGGCGACTGTA
349347-349598      206  TTTTTT...ATGACCTTTTTTTG........CCCCCATTTGACGCTTTGGCGACTGCA
1732017-1732183    145  TTTTT....ATAACCTTATTTTG.........CTCC......................
2316942-2317180    203  TTTTTTT..ATGACTTTCTTTTG........CCCCCCTTTGGCAGTT...........
662519-662768      206  TTTTT....ATGACTTTGTTTTG.........CCCCCTTTGAGAGTTCACCGACTGTA
2366989-2367235    203  TTTTT....ATGACTTTGTTTTG.........CCCCTTTTGAGAGTTCACCGACTGTA
29444-29711        211  TTTTTTTTATTGGCTGTATTTCGCTCCCCCCCCCCCTCTTGATAGTTTGCGGACTATA
17707-17952        202  TTTTT....ATGACTTTATTTTG.........CCCCCTTTGAGAGTTCGGGGACTGTA
```
