# Supplementary figures and images for: DNA transposons have colonized the genome of the giant virus Pandoravirus salinus
Source: BMC Biol. 2015 Jun 12;13:38. doi: 10.1186/s12915-015-0145-1 (PMC4495683; doi:10.1186/s12915-015-0145-1)

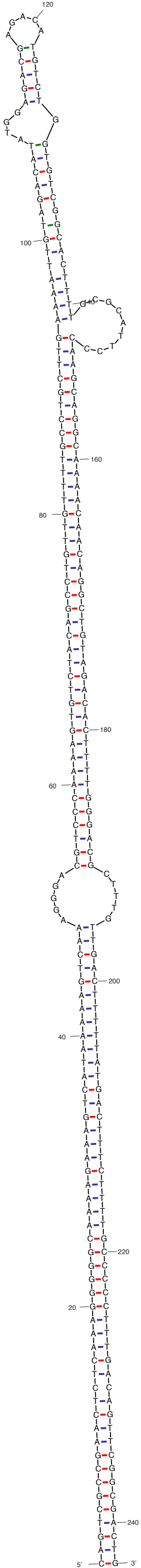

*dG = -110.63 MITE\_consensus*

Supplement: Additional file 2: Figure S2. — The secondary structure of the P. salinus miniature inverted-repeat transposable element (MITE) consensus sequence [Repbase ID: Submariner_Ps1] predicted by mFold. [file 12915_2015_145_MOESM2_ESM.pdf]

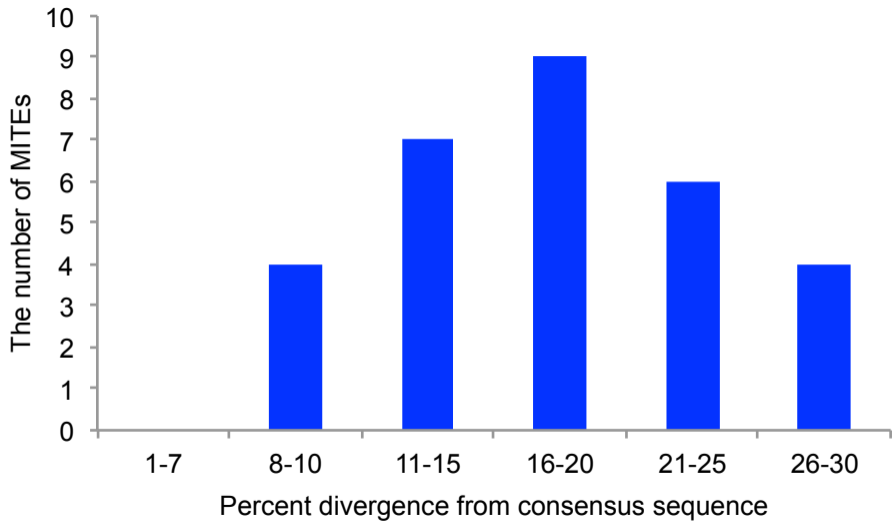

Supplement: Additional file 4: Figure S4. — Frequency histogram showing pairwise divergences between individual MITE insertions in P. salinus and the miniature inverted-repeat transposable element (MITE) consensus sequence. [file 12915_2015_145_MOESM4_ESM.pdf]

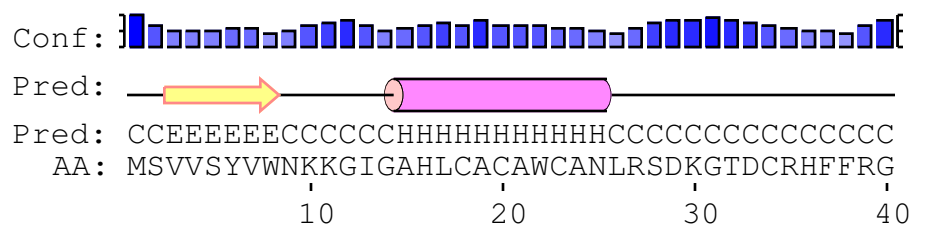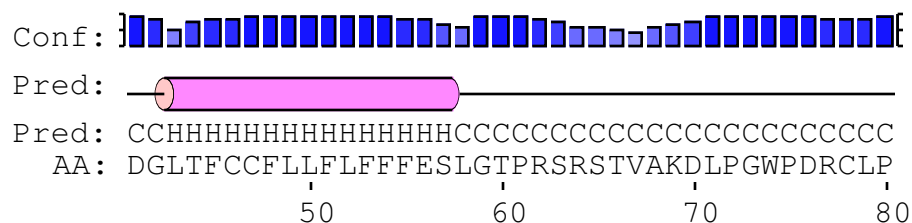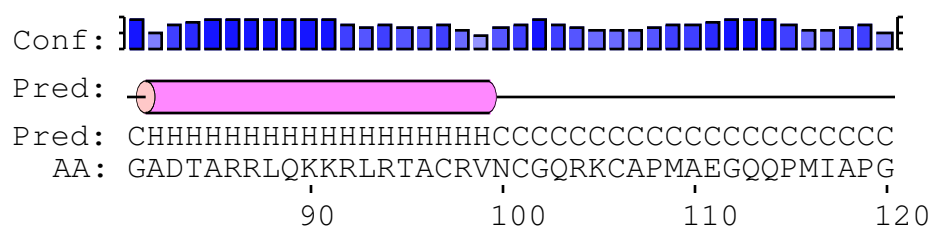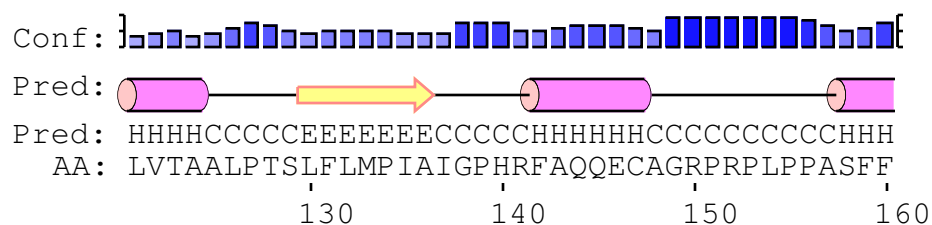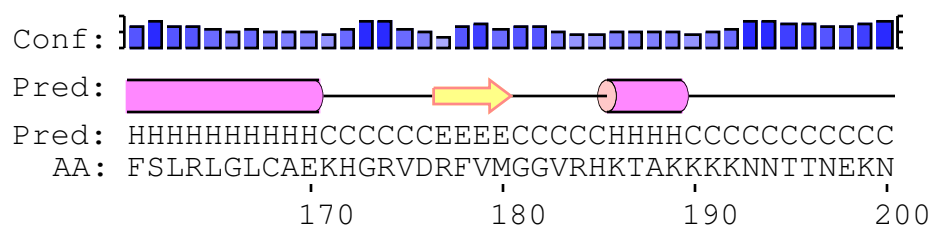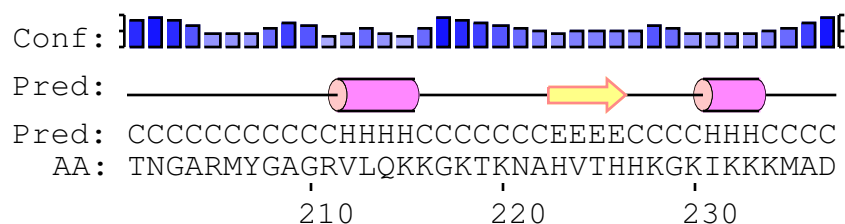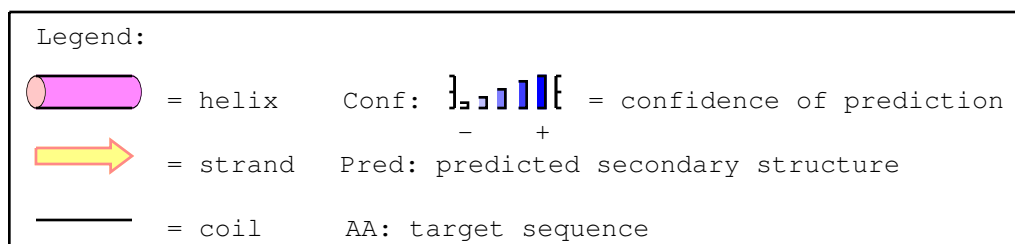

Supplement: Additional file 10: Figure S8. — Predicted secondary structure of the hypothetical protein encoded by P. salinus gene Ps_1377, one of the predicted P. salinus open reading frames that includes miniature inverted-repeat transposable element (MITE) sequence. In this example, the first 60 amino acids of the predicted protein are MITE-derived and form three stable secondary structures: one β-strand and two α-helices. Secondary structure prediction was done using PSIPRED. [file 12915_2015_145_MOESM10_ESM.pdf]
