# Supplementary material for: DNA transposons have colonized the genome of the giant virus Pandoravirus salinus
Source: BMC Biol. 2015 Jun 12;13:38. doi: 10.1186/s12915-015-0145-1 (PMC4495683; doi:10.1186/s12915-015-0145-1)
Supplement: Additional file 3: Figure S3. — Pairwise alignment of a site with a miniature inverted-repeat transposable element (MITE) insertion (bottom sequence) and its paralogous empty site (top sequence) in P. salinus. Target site duplications are underlined in red. [file 12915_2015_145_MOESM3_ESM.pdf]

```
                                1
gb|KC977571.1|:895224-895342   ACGGTGGGCAGCGGCTAGCCGACCG GCTAAAAACATGCGAATTCCACTGT
gb|KC977571.1|:17649-18010     ACGGTGGGCAACGGCTAGGTGGCCG GCT-AAAACACGCGAATTCCACTGT

                               51
gb|KC977571.1|:895224-895342   CGACGTCCC---------------- -------------------------
gb|KC977571.1|:17649-18010     CCACGTCCCTACAGTCCCCGAACCC TCAAAGGGGGCAAAATAAAGTCATA

                              101
gb|KC977571.1|:895224-895342   ------------------------- -------------------------
gb|KC977571.1|:17649-18010     AAAAGTCAAAGGGACGTCCCAAAAA TGTCTACAGCCTGTTGTTTCGTCTG

                              151
gb|KC977571.1|:895224-895342   ------------------------- -------------------------
gb|KC977571.1|:17649-18010     CTTAAAAATTGTAGGCATATGGGGG TGAGACATGTCTGATGTCGGCACTT

                              201
gb|KC977571.1|:895224-895342   ------------------------- -------------------------
gb|KC977571.1|:17649-18010     TCGCGCACTCCCAAGCGGACAAAAC AACAGGCTGTAGACATTTTGGGACG

                              251
gb|KC977571.1|:895224-895342   ------------------------- -------------------------
gb|KC977571.1|:17649-18010     CCCCTTTGACTTTTTATGACTTTAT TTTGCCCCCTTTGAGAGTTCGGGGA

                              301
gb|KC977571.1|:895224-895342   ---TACTATGTCAGGATGAATCCAC AATTTTTAGCCGCTCGGCTGGCCGT
gb|KC977571.1|:17649-18010     CTGTACCACGTCAGGATCAATCCAC AATTTTTAGCCGTTCGTCTAGCCGT

                              351
gb|KC977571.1|:895224-895342   TGCCCAGCATTAG
gb|KC977571.1|:17649-18010     TGCCCAGCATTAG
```
