# Supplementary material for: DNA transposons have colonized the genome of the giant virus Pandoravirus salinus
Source: BMC Biol. 2015 Jun 12;13:38. doi: 10.1186/s12915-015-0145-1 (PMC4495683; doi:10.1186/s12915-015-0145-1)
Supplement: Additional file 5: Figure S5. — Pairwise alignment of a site with a DNA transposon insertion (bottom sequence) and its paralogous empty site (top sequence) in A. castellanii. Target site duplications are underlined in red. [file 12915_2015_145_MOESM5_ESM.pdf]

```
                                   1
gb|AEYA01001963.1|:3377-3491       CAACCGTGAATTGGGCGATTGGTTGGCGGACATTCCCAGTTTGATCCCAT
gb|AEYA01001964.1|:92173-93899     CAACCGTGAATTGGGCGATTGGTTGGCGGACATTCCCAGTTTGATCCCAT

                                  51
gb|AEYA01001963.1|:3377-3491       TGGCACCTGGCGCACGGAAC------GTTTCGAAC---------------
gb|AEYA01001964.1|:92173-93899     TGGCACCTGGCGCACGGAACAAAATAGTTTCGAACTACAGTCGTCGGAAA

                                 101
gb|AEYA01001963.1|:3377-3491       --------------------------------------------------
gb|AEYA01001964.1|:92173-93899     GTCAAAGGGGGCAAAAGCCCTTCCCAACTCCGGCATTTTTTTCGTCTTGC

                                 151
gb|AEYA01001963.1|:3377-3491       --------------------------------------------------
gb|AEYA01001964.1|:92173-93899     CTGCCCCGTTCACACCAACGCCAGTAAGACACCCGAGCGAGGGCGCAGGA

                                 201
gb|AEYA01001963.1|:3377-3491       --------------------------------------------------
gb|AEYA01001964.1|:92173-93899     TCGGGGCGCTGAAGATTGCAAGTGAAAAGGTTCTTAATTGATTTCATCAT

                                 251
gb|AEYA01001963.1|:3377-3491       --------------------------------------------------
gb|AEYA01001964.1|:92173-93899     ATATATCATTTACAAACAGTTCATCTTCCATCTTTTCTACCTTGGCGATC

                                 301
gb|AEYA01001963.1|:3377-3491       --------------------------------------------------
gb|AEYA01001964.1|:92173-93899     GCTTTCTTCCACATCTCTGGAGTGACGTGCTCCACACCTTCCTTTGTCAA

                                 351
gb|AEYA01001963.1|:3377-3491       --------------------------------------------------
gb|AEYA01001964.1|:92173-93899     TCGTTCGATCTCCCCCATCGCAAATTGCCGTCGGTTTCGTGCTACATAGC

                                 401
gb|AEYA01001963.1|:3377-3491       --------------------------------------------------
gb|AEYA01001964.1|:92173-93899     CCTTCACGATTCCCCACATGTTTTAGATCGGGTTGAAGTCGGAATGGTAG

                                 451
gb|AEYA01001963.1|:3377-3491       --------------------------------------------------
gb|AEYA01001964.1|:92173-93899     GGGGGGAGGAAGATGATGTTGTGTCCATAACGGCGTGCTATTTCGTAGAT

                                 501
gb|AEYA01001963.1|:3377-3491       --------------------------------------------------
gb|AEYA01001964.1|:92173-93899     GGCTGGAGGTGATGCCTCGTGGGCCTCTTTTGCAGTGTGGTAGAGTTTCT

                                 551
gb|AEYA01001963.1|:3377-3491       --------------------------------------------------
gb|AEYA01001964.1|:92173-93899     CTCGTGTCCATCCTGCTTCCCATTCGATTTGGTGGTGCGAGAGGTAGCGT

                                 601
gb|AEYA01001963.1|:3377-3491       --------------------------------------------------
gb|AEYA01001964.1|:92173-93899     TGGAGTTGGGATTTCTGAAGATTACTGAAGGTTCGCCCCTCGAAGATCTC

                                 651
gb|AEYA01001963.1|:3377-3491       --------------------------------------------------
gb|AEYA01001964.1|:92173-93899     TTTCTCTGCGACACTCACCATCTTGACCTTGTGGTAGCCAGCATTATCCA

                                 701
gb|AEYA01001963.1|:3377-3491       --------------------------------------------------
gb|AEYA01001964.1|:92173-93899     TTACAAAGGTTGATGGCTTGGGAATGGCACAGCAGACCTTCTCCTCAAAC

                                 751
gb|AEYA01001963.1|:3377-3491       --------------------------------------------------
gb|AEYA01001964.1|:92173-93899     CATTTCACAAAAATCAAGTGATCAACATTCTCATGGTAATCAGTGGTCTT

                                 801
gb|AEYA01001963.1|:3377-3491       --------------------------------------------------
gb|AEYA01001964.1|:92173-93899     TGACTTTGACTTCTTCACAACCCACATCTGCTGTGCTCCTGGTATCCATC

                                 851
gb|AEYA01001963.1|:3377-3491       --------------------------------------------------
gb|AEYA01001964.1|:92173-93899     CTTTCTCTCCTCCAGCGTGCAGAAGTATGAGTCACTGACCCTTCCCTGCT

                                 901
gb|AEYA01001963.1|:3377-3491       --------------------------------------------------
gb|AEYA01001964.1|:92173-93899     GGTGTGCCAACTGCATTCCCTTCCAGTCAGAGGAACCAGGTGTAGTTTGG
```

```
                                 951
gb|AEYA01001963.1|:3377-3491     --------------------------------------------------
gb|AEYA01001964.1|:92173-93899   CACATGGTTCTGATGGATGTAGGTTTCATCAAGGAAGTAGGTAGGCCGCT

                                1001
gb|AEYA01001963.1|:3377-3491     --------------------------------------------------
gb|AEYA01001964.1|:92173-93899   GATGTCCTTCTGCCCTCTCCATCACTCTCTGGTCAATGTACACTCACCGC

                                1051
gb|AEYA01001963.1|:3377-3491     --------------------------------------------------
gb|AEYA01001964.1|:92173-93899   CAAGCCTGTACTCTCTTGCTCTCAAACAACTCTCCTTTCTTCTTCACTTC

                                1101
gb|AEYA01001963.1|:3377-3491     --------------------------------------------------
gb|AEYA01001964.1|:92173-93899   ACCCCATTCAAATCCCATCTTGCAAATGTAGTGGCACAGACACTCTTTGT

                                1151
gb|AEYA01001963.1|:3377-3491     --------------------------------------------------
gb|AEYA01001964.1|:92173-93899   TGACTTCAACTCCTTTCTCCTCCTTCAACCACCACACTAGGCGAGCCATC

                                1201
gb|AEYA01001963.1|:3377-3491     --------------------------------------------------
gb|AEYA01001964.1|:92173-93899   AAAGCCAGACTTCCTGCTCTATTCCAATCTCAAATCTGCTGTGCTGCTTC

                                1251
gb|AEYA01001963.1|:3377-3491     --------------------------------------------------
gb|AEYA01001964.1|:92173-93899   CTTTAATGTCTCATGCTCAATCTTTGGACCCTTGAGCCACTTCCATTGTT

                                1301
gb|AEYA01001963.1|:3377-3491     --------------------------------------------------
gb|AEYA01001964.1|:92173-93899   TCTTCTTCTTCTCCAGCATCTCCTTCTCCACATAAACCTCAGTAATCAGT

                                1351
gb|AEYA01001963.1|:3377-3491     --------------------------------------------------
gb|AEYA01001964.1|:92173-93899   TCTTGCATCTTCCTCCAACTTATTCCTACCCACTTGCACACCACTTCCCT

                                1401
gb|AEYA01001963.1|:3377-3491     --------------------------------------------------
gb|AEYA01001964.1|:92173-93899   GGTGTTGTTCACCCCCAGCAAAGATGCATGGGCCCAAAGTCACAAGATGA

                                1451
gb|AEYA01001963.1|:3377-3491     --------------------------------------------------
gb|AEYA01001964.1|:92173-93899   TGTTGATCAAATCTTGGGATAGTGGATGCCCAGAGACTGAGATGGAGTAG

                                1501
gb|AEYA01001963.1|:3377-3491     --------------------------------------------------
gb|AEYA01001964.1|:92173-93899   TTTGCTAACACCTCCAACAACTGTTGCTTCTCTTCACCTTGCACCCTCTT

                                1551
gb|AEYA01001963.1|:3377-3491     --------------------------------------------------
gb|AEYA01001964.1|:92173-93899   CTTTGTTTTCTCCTCATCCTCCAGCATCTTCAATAAATTTGATTGTTGCT

                                1601
gb|AEYA01001963.1|:3377-3491     --------------------------------------------------
gb|AEYA01001964.1|:92173-93899   GAGCAGAAAACAGTAGGCACTGGTTGTTCTTACTCTCACCTATGTTGACC

                                1651
gb|AEYA01001963.1|:3377-3491     -----------------------------------------TAAAACAGC
gb|AEYA01001964.1|:92173-93899   AAATATGACTTTTTTTGCCCCCTTTGAGTGTTTGGGGACTGTAACACAGC

                                1701
gb|AEYA01001963.1|:3377-3491     AAAGGGGCACAACTACTCCCTTCCCTC
gb|AEYA01001964.1|:92173-93899   AAAGGGGCACAACTACTCCCTTCCCTC
```
