# Supplementary material for: DNA transposons have colonized the genome of the giant virus Pandoravirus salinus
Source: BMC Biol. 2015 Jun 12;13:38. doi: 10.1186/s12915-015-0145-1 (PMC4495683; doi:10.1186/s12915-015-0145-1)
Supplement: Additional file 6: Figure S6. — Multiple sequence alignment of (1) the putative transposase sequences from Submariner_Ac1 and Submariner_Ac2, (2) the four DNA transposase hits we obtained from Repbase using Submariner_Ac1 as a query (Mariner-1_AP, Mariner-2_AP, Mariner-3_AP, and Mariner44_CB), (3) two representative transposases from each of five well-established Tc1/mariner clades (Fot1, Pogo, Tc1, Gizmo, and Mogwai), and (4) the five hits obtained from the NCBI non-redundant protein database (nr) using Submariner_Ac1 as a query (four bacterial sequences — Beggiatoa sp. PS, gi|152068700; Deltaproteobacterium NaphS2, gi|300441029; Candidatus Magnetoglobus multicellularis str. Araruama, gi|571786598; and Desulfobacula sp. TS, gi|667676338, and one uncultured archaeal sequence, GZfos18F2, gi|52548731). Sequences are identified by their GenBank accession numbers or Repbase IDs, if applicable, which correspond to the nucleotide sequences from which the transposase amino acid sequences were deduced. The multiple alignments were generated by PSI-Coffee, an aligner within the T-Coffee multiple alignment package that aligns distantly related protein sequences using homology extension [83, 84]. Red arrows indicate the DDE amino acid triad that coordinates metal ion (Mg2+) binding during catalysis of typical cut-and-paste transposition. [file 12915_2015_145_MOESM6_ESM.pdf]

```
T-COFFEE, Version_11.00.8cbe486 (2014-08-12 22:05:29 - Revision 8cbe486 - Build 477)
Cedric Notredame
CPU TIME:2 sec.
SCORE=657
*
 BAD AVG GOOD
*
gi|152068700        :  70
gi|300441029        :  74
gi|571786598        :  70
gi|667676338        :  71
gi|52548731         :  71
Mariner-1_AP        :  71
Mariner-2_AP        :  71
Mariner-3_AP        :  71
Mariner44_CB        :  67
Submariner_Ac1      :  69
Submariner_Ac2      :  71
Cirt2_CA-Fot1       :  51
Fot1_FO-Fot1        :  55
Gizmo1_EI-Gizmo     :  56
Gizmo2_EI-Gizmo     :  59
MOGWAI1_EI-Mogw     :  57
MOGWAI2_EI-Mogw     :  56
Mariner-1_NV-Po     :  50
Mariner2_MT_DNA     :  48
BARI_DM-Tc1         :  57
FB4_DM-Tc1          :  54
cons                :  65


gi|152068700        MSQQGKT--LSIEQK-----------------------------------Q------------
gi|300441029        ---------------------------------------------------------------
gi|571786598        MSFQGKE--FTQGMK-----------------------------------Q------------
gi|667676338        MSFQGVE--FTPEMR-----------------------------------K------------
gi|52548731         MTQQGKA--ILFEQK-----------------------------------Q------------
Mariner-1_AP        MI-------------------------------------------------------------
Mariner-2_AP        KRQI-----------------------------------------------------------
Mariner-3_AP        MI-------------------------------------------------------------
Mariner44_CB        MFQTELR--HM-RIG-----------------------------------Q------------
Submariner_Ac1      REV------------------------------------------------------------
Submariner_Ac2      V--------------------------------------------------------------
Cirt2_CA-Fot1       MAKAI----------------------------------------------------------
Fot1_FO-Fot1        MPVYS----------------------------------------------------------
Gizmo1_EI-Gizmo     IQNDNCK-SLLTKLR-----------------------------------K------------
Gizmo2_EI-Gizmo     IEKGGI--NFP----------------------------------------------------
MOGWAI1_EI-Mogw     MKKFDWAFPLTMECRKGLSMDLQLNFSFGAQATPFNICKVPPKPTFFETEPEITNETKYFENP
MOGWAI2_EI-Mogw     MYGMTAS--------------------------PFNIGHVKKEEIIEELKEQEFEYFPIFKIP
Mariner-1_NV-Po     MAFHVRF--NLLFL-----------------------AMA-------KRNR------------
Mariner2_MT_DNA     MNVLKVF--KPYL--------------------------------------------------
BARI_DM-Tc1         KLDLII---FL----------------------------------------------------
FB4_DM-Tc1          KNFIL----------------------------------------------------------

cons


gi|152068700        ------------------------------------------------------FII--NLKW
gi|300441029        ---------------------------------------------------------------
gi|571786598        ------------------------------------------------------LVI--NLKQ
gi|667676338        ------------------------------------------------------MVV--NVKH
gi|52548731         ------------------------------------------------------YII--NLKK
Mariner-1_AP        --------------------------------------------------------V--NLFK
Mariner-2_AP        -------------------------------------------------------II--NAYK
Mariner-3_AP        --------------------------------------------------------V--NLYK
Mariner44_CB        ------------------------------------------------------SLC--DIDT
Submariner_Ac1      ---------------------------------------------------------------
Submariner_Ac2      ---------------------------------------------------------------
Cirt2_CA-Fot1       ---------------------------------------------------------------
Fot1_FO-Fot1        ---------------------------------------------------------------
Gizmo1_EI-Gizmo     -----------------------------------------------------GDSI--LPKN
Gizmo2_EI-Gizmo     -------------------------------------------------------------KN
MOGWAI1_EI-Mogw     NEKDPIFIRNAFLASKHIDQHFEMYNARMKEISKQYYEENDIHEVLFPRQIVPSTQLNLKRKK
MOGWAI2_EI-Mogw     SEDIEFEYCGKYLISKLLDDHFNTYNDTIKKMSKTNTLTIEPLENLFPTQLVSSTSI--KLNK
Mariner-1_NV-Po     ------------------------------------------------------TCI--DLNR
Mariner2_MT_DNA     ---------------------------------------------------------------
BARI_DM-Tc1         -------------------------------------------------------------LQ
FB4_DM-Tc1          ---------------------------------------------------------------

cons


gi|152068700        SYDVE-----RN-----------------------------IGP-------------------
gi|300441029        ---------------------------------------------------------------
gi|571786598        FYDTE-----RI-----------------------------KNG-------------------
gi|667676338        YFDNL-----KL-----------------------------TPN-------------------
gi|52548731         SFDLE-----RK-----------------------------KGS-------------------
Mariner-1_AP        SKMIQ-------------------------------------QP-------------------
Mariner-2_AP        SEMNS-------------------------------------DP-------------------
Mariner-3_AP        NKMAL-----QA----------------T-X----------DVP-------------------
Mariner44_CB        SIFPK-----TPTQKHTRNSGEEIMRNVSSSVGIIQEKLGSDCR-------------------
Submariner_Ac1      ---------------------------------------------------------------
Submariner_Ac2      ---------------------------------------------------------------
Cirt2_CA-Fot1       --------SDIKNG-------------L-----------------------------------
Fot1_FO-Fot1        ---------------------ADDLEN---AIA------------------------------
Gizmo1_EI-Gizmo     HYNKK-----SRVT--------------PYQ----------VXVGRLLENDSTTKVHQMREYI
Gizmo2_EI-Gizmo     HYRRK-----SRVI--------------PFQ----------NLVQKILEKDSTESVSNIRRHI
MOGWAI1_EI-Mogw     NYNER-----KNDL--------------YTQ----------IYI-------------------
MOGWAI2_EI-Mogw     NENEK-----RKLL--------------YVK----------ILI-------------------
Mariner-1_NV-Po     KIEL-------LN-----------------------------YA-------------------
Mariner2_MT_DNA     ---------------------------------------------------------------
BARI_DM-Tc1         LLNKMPKTKELTVE-------------ARAG----------IVA-------------------
FB4_DM-Tc1          -----------------VGRGQHYSQGKRMLILKLRKE-------------------------

cons


gi|152068700        TTSTKGSV-GRVAKGLGIGKRTVE----SILAQYQKEGQ-TLI-ET-EPKPRGKPPFSLDASL
gi|300441029        ---------------------------------------------------------------
gi|571786598        KK-ASWAI-EQTAKGLKIGEATVR----RVMAEYNKNKQ-DIP-YS-LPKPKGKPDFSITNDL
gi|667676338        TF-GSPAT-QLAASALGISESTVK----VIMAAYNKKGE-DGL-DWSKSQQRGRPAYALESGV
gi|52548731         VVSTKDSI-GRITRCLDIGRRTVE----NIISEYNKNGQ-ILI-KT-PPKTRGKPPLKVSGDL
Mariner-1_AP        TLKVKEVA-MIISKELGIGKNTIQ----STIAEYKNKKT-VSS-PN-KSKIRATYKQKVDDFE
Mariner-2_AP        TKSLRIIR-QELSKELGIGATTIS----TTITEYNNTKK-VIS-PC-KKRVKTSLLETFEEFE
Mariner-3_AP        KLTAKEMI-KKISEESGIGQRTVS----VTLSEYRNKKT-VTS-PN-KTKIRPKVTDKVDEFD
Mariner44_CB        FTMFSNTA-KATAEMCGLSRKTVI----RLTPERDLPQK-KLLVTMGKKERCRRYASQLDFVK
Submariner_Ac1      ------------VCKWVGISWRKMQ----ELITEVYVEKE-MLE-KK-KKQWKWLKGPKIEHET
Submariner_Ac2      -------------AKWVGISESKMA----QLIAEVYEVGE-VLK-KK-LKKCEWTKEGKIPNSL
Cirt2_CA-Fot1       ----LLSY-RKAAKKYNLCHETIR----KRMNGVLPKQIAHRG-K-Q----------------
Fot1_FO-Fot1        DFKNGVSL-KTAAKKNGLPPSTLR----GRLTGAQSRQV-ARQ-EQ---------LRLTTDQ
Gizmo1_EI-Gizmo     KGMMVMDDIVNVEDIVEIREETFK----EIVE-----HP-EII--------------------
Gizmo2_EI-Gizmo     KYITAKSNDRNLE---DVSLVTKQ----EFEAGYHRRFN-EPL--------------------
MOGWAI1_EI-Mogw     YYSDCKNY-NRTAKQFKLPPSTIH----NIIERVKNGVS-LTG-N-XKGRKSGHN-YKFDYNT
MOGWAI2_EI-Mogw     HYLGCRNA-HNTANVFNVCPQTVI----NIYRRAMNNED-LLG-Q-QRGPKVGQN-NKVDYHT
Mariner-1_NV-Po     KDNHMHGV-RVIAAKYGIGKTQVS----KILQNKE----------------------------
Mariner2_MT_DNA     ------TH-EMASHLKGVAKSTMSDQIRKELCEYKRDNP------------------------
BARI_DM-Tc1         RFKAGTPA-AKIAEIYQISRRTVY----YLIKKFDTVGT-LKN-K-KR---SGRK-PVLDQRQ
FB4_DM-Tc1          ----GKTY-KDIQKTLKCSAKMVS----NAIKY-----------KWKPENRGT-KHKTTDIED

cons


gi|152068700        IPPIRN-Y--------------VRYMNKIGQ-YLSIRRIR-----SWLIQ-K-----------
gi|300441029        -PIVRK-F--------------IRTENLGGR-RVSIGRVC-----KFLSS-K-----------
gi|571786598        LPHVRK-H--------------IRSQNLIGQ-HASLETVS-----EYLKSID-----------
gi|667676338        ISHVRQ-F--------------IRRANRNGD-QVNVEIVR-----RYMRD-E-----------
gi|52548731         IPFIRQ-H--------------IRSANLQGQ-HISVRNVR-----SWLLK-E-----------
Mariner-1_AP        RDAIRR-K--------------VHEFWFRKQ-LPTLDKIL-----TAVNE-D-----------
Mariner-2_AP        RNVVRR-H--------------VHSFWFKRE-IPTVDKIF-----QVVSD-D-----------
Mariner-3_AP        QNAIRQ-K--------------VHQFWHNHQ-IPTLNKIL-----TAVNE-D-----------
Mariner44_CB        RVRIVA---------------KIHSLWKEGR-DVTVSDLW-----TFAKD-E-----------
Submariner_Ac1      LKEAAQ---------------QID-WNRAGS-LALMARLV-----WWLKE-E-----------
Submariner_Ac2      LQVMVD---------------QIE--WNNGT-LALLPKLQ-----QWLQS-E-----------
Cirt2_CA-Fot1       ---------LFTPQQEKEIVKWIIESDEAGN-GRSRHDIV-----EYAEL-FL---------LL
Fot1_FO-Fot1        EDDLER---------------WILRQEKLGH-APTHAQVR-----TIVRS-VL---------AR
Gizmo1_EI-Gizmo     VGIDKKME------------------KKNK-IVPSITSIT-----NFMRG-K-----------
Gizmo2_EI-Gizmo     T--PES-L------------------NLADSLIPSVSSIT-----NFMRG-K-----------
MOGWAI1_EI-Mogw     ITMILE-E------------------ISANK-HTTLKLIT-----KHVNE-KKRDEWMEAIAR
MOGWAI2_EI-Mogw     LTLIID-E------------------LRKNN-QITLKNLT-----KYVNE-HR-LEWMKEVAK
Mariner-1_NV-Po     ---------------------------------------------------------------
Mariner2_MT_DNA     --------------------------------ASTQKDLQ-----RWLEG-K-----------
BARI_DM-Tc1         CRQILG-VVAKNPS-ASPVKIALESKNTIGK-QVSSSTIR-----RR----------------
FB4_DM-Tc1          R-----------------------------R-IVSYSKVYRFASFRDIKS-E-----------

cons


gi|152068700        ----------------HK-IDIPTMTLCRFLV-------------------------------
gi|300441029        ----------------HG-IDVPKMTLWRALN-------------------------------
gi|571786598        ----------------PK-YNFSTTTSWRTLH-------------------------------
gi|667676338        ----------------LH-CDVAHTTLWRALQ-------------------------------
gi|52548731         ----------------FN-ADIPIMTLWRSLQ-------------------------------
Mariner-1_AP        ----------------PDLNTYKRSTLHLLIH-------------------------------
Mariner-2_AP        ----------------DSLPIISRTNLFRLLK-------------------------------
Mariner-3_AP        ----------------DSLPSFSKISLHRVLK-------------------------------
Mariner44_CB        ----------------LK-FQRGRSVFLYVLR-------------------------------
Submariner_Ac1      ----------------KG-VEVNKECLCHYIC-------------------------------
Submariner_Ac2      ----------------HR-RNVSERQMRHYIS-------------------------------
Cirt2_CA-Fot1       -------D-------RQL-ASIGGSWFERFKK-------------------------------
Fot1_FO-Fot1        H--------------GDH-APLGRKWTTRFVE-------------------------------
Gizmo1_EI-Gizmo     ----------------TG-VDEDRE-----LP-------------------------------
Gizmo2_EI-Gizmo     ----------------TG-NEMGRT-----FP-------------------------------
MOGWAI1_EI-Mogw     YESYSMRDEHEIKEMLKQ-KKVSLSGVHRILK-------------------------------
MOGWAI2_EI-Mogw     IEMELGADENVINKMFKQ-SKLSKSTISILVQ-------------------------------
Mariner-1_NV-Po     -----------------------------------TILASYENNNGHIKI--KRIRTGKYANV
Mariner2_MT_DNA     ----------------FQ-LKVSQGTISNTLKRSNDYLSAEIE-KGRAEIKRHKP--AKYPDM
BARI_DM-Tc1         ------------------------------LK-------------------------------
FB4_DM-Tc1          ----------------LN-LGISDVTIRRRLL-------------------------------

cons


gi|152068700        -------------------------------------------------------RIGVVYGT
gi|300441029        -------------------------------------------------------RWGFSHGE
gi|571786598        -------------------------------------------------------RWGFTYGK
gi|667676338        -------------------------------------------------------RWGFEFGT
gi|52548731         -------------------------------------------------------RVGFVYGK
Mariner-1_AP        -------------------------------------------------------DLNFVYVK
Mariner-2_AP        -------------------------------------------------------EMDFKYSK
Mariner-3_AP        -------------------------------------------------------HLNFEYVR
Mariner44_CB        -------------------------------------------------------GLGFVYRQ
Submariner_Ac1      -------------------------------------------------------KMGFEWGE
Submariner_Ac2      -------------------------------------------------------KVGFQWVE
Cirt2_CA-Fot1       -------------------------------------------------------RHKEI-HV
Fot1_FO-Fot1        -------------------------------------------------------RHPALKTK
Gizmo1_EI-Gizmo     -------------------------------------------------------ILSF--KR
Gizmo2_EI-Gizmo     -------------------------------------------------------ILSF--KK
MOGWAI1_EI-Mogw     -------------------------------------------------------RLGVTLKL
MOGWAI2_EI-Mogw     -------------------------------------------------------KLGWSLKK
Mariner-1_NV-Po     NDAVWDWYQMCRNFNIPISGTMIQEEVMIIAERLGLNE----FTGSNGWLEKFKRQHNIGQMA
Mariner2_MT_DNA     EKVVYEWFLQHQ-ERVNITGELILQKARD-TMKLLYPHDDSDFNFSTGWLGKFKHRHGIKSFR
BARI_DM-Tc1         -------------------------------------------------------EADFKTYV
FB4_DM-Tc1          -------------------------------------------------------NQNFSARS

cons


gi|152068700        -----G-KRRSSLKETDKIII-------ARR-EYL-RQKIKNRRK------DGTKKRPEVYLD
gi|300441029        -----G-RRRNSLKEQDRIIF-------ARR-EYL-RAKLANRNP------DGTLKRPEVYLD
gi|571786598        -----E-QRRSALKERDNVIL-------NRR-RYL-RQKRLNRNS------DGTFKRVEVYLD
gi|667676338        -----G-VRSAQLKESERIII-------LRR-QYL-RQKLANRDD------NGQIIRPEVYID
gi|52548731         -----N-KRRCALKEKEYVVT-------ARR-KYL-RKKMANRKA------DGTLIRPEVYLD
Mariner-1_AP        -----R-GRNSALIERDDIVL-------WRT-KYI-EDIRKYRA----------QRRTIYYLD
Mariner-2_AP        -----R-GRNSALTEKPKFCV-------ARR-RFL-EQLREYRN----------EGRHLYYLD
Mariner-3_AP        -----K-SRNSVLIERNDIVC-------WRR-NYL-ETIKNYRQ----------LGRQIYYLD
Mariner44_CB        ------KEFNPIVSERPDIVH-------KRM-LYL-QLKKEWDS----------KNALYGSFD
Submariner_Ac1      ------VKKKGELFESKRVQA-------W-R-VYI-DQRVMERAE--------GHQRPTYFLD
Submariner_Ac2      ------VKKRGEAFDSLCVQD-------LKK-AYI-QLHMSKRMAQERGGREGEEEWPVYFLN
Cirt2_CA-Fot1       VQGRKISSLRVKAATPDQIKE-------YF-HKYD-LIVRQHQI----------PNENIFNYD
Fot1_FO-Fot1        ------LGR-R-T-DWERVNAATPANIKRLF-DVY-ETVDWI------------PPERRYNAD
Gizmo1_EI-Gizmo     -----E-TTRGAESNSEVNKD-------KRI-NAI-QKLYARMC----------EGYSWVCLD
Gizmo2_EI-Gizmo     -----E-TTRGAVANTPDNKE-------KRV-EAI-EKLVGMMH----------QGYTWVCVD
MOGWAI1_EI-Mogw     -----V-KKEQFNRNVKVRIE-------QRK-MYA-DHIEYLNY----------FQFVYAYID
MOGWAI2_EI-Mogw     -----V-KRNIFDKNSEIRIQ-------QRI-VYA-EHIDFIEV----------FKYRVAYID
Mariner-1_NV-Po     -----V-SGEEAGVNPETVES-------WKE-RAR-EITRGW------------DAKNVWNMD
Mariner2_MT_DNA     -----R-FGESGSVDVQDMEQ-------KLV-SIR-EKIDQF------------PMKDVFNMD
BARI_DM-Tc1         ------VRKTIEITPTNK--T-------KRLRFAL-EYVKKPLD----------FWFNILWTD
FB4_DM-Tc1          ------PRKVPLP-SP-RHIK-------ARL-SLAKTYLNWPVS----------KWRNILWTD

cons                                                                               :
```

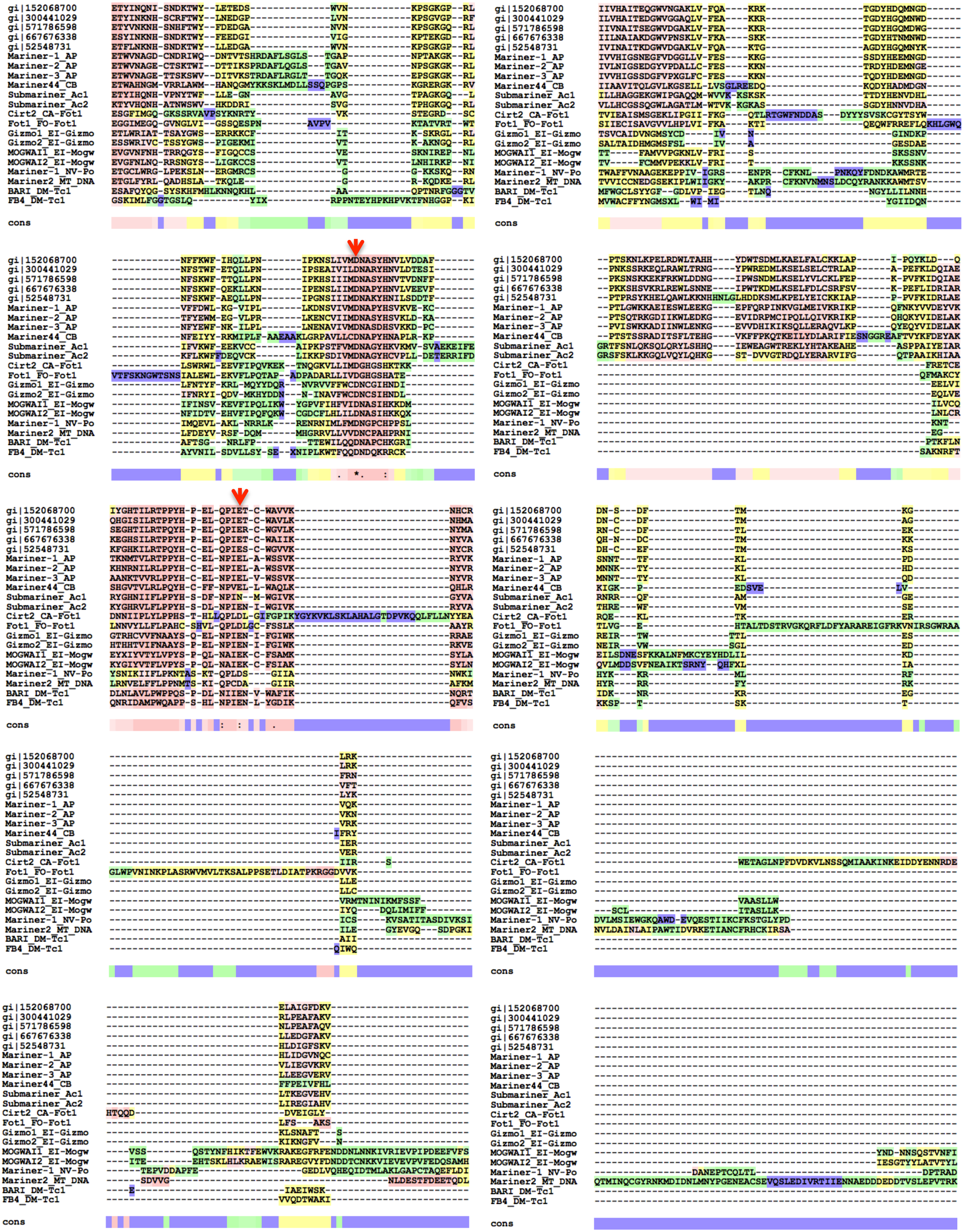
gi|152068700
gi|300441029
gi|571786598
gi|667676338
gi|52548731
Mariner-1_AP
Mariner-2_AP
Mariner-3_AP
Mariner44_CB
Submariner_Ac1
Submariner_Ac2
Cirt2_CA-Fot1
Fot1_FO-Fot1
Gizmo1_EI-Gizmo
Gizmo2_EI-Gizmo
MOGWAI1_EI-Mogw
MOGWAI2_EI-Mogw
Mariner-1_NV-Po
Mariner2_MT_DNA
BARI_DM-Tc1
FB4_DM-Tc1
cons

```
gi|152068700       --------------------------------TNKTCQ-------------------------
gi|300441029       --------------------------------TTSTCK-------------------------
gi|571786598       --------------------------------KAETCR-------------------------
gi|667676338       --------------------------------TEKTCR-------------------------
gi|52548731        --------------------------------TKSTCQ-------------------------
Mariner-1_AP       --------------------------------TPEMWE-------------------------
Mariner-2_AP       --------------------------------DADMWK-------------------------
Mariner-3_AP       --------------------------------TPDMWK-------------------------
Mariner44_CB       --------------------------------GEKPCQG------------------------
Submariner_Ac1     --------------------------------TPEMWK-------------------------
Submariner_Ac2     --------------------------------TPEMWK-------------------------
Cirt2_CA-Fot1      -------------------------L------RGTPDE-------------------------
Fot1_FO-Fot1       --------------------------------SSPSSRLSIRKAAAALDKVAIELAMKDREIE
Gizmo1_EI-Gizmo    -------------------------I------ET---P-------------------------
Gizmo2_EI-Gizmo    -------------------------I------QP---A-------------------------
MOGWAI1_EI-Mogw    SRNDVLHVLTYDPFTIKNEK---SFT------NC---E-------------------------
MOGWAI2_EI-Mogw    NRVNAVHVLSDYI--IQVDDTNCTPS------AQ---I-------------------------
Mariner-1_NV-Po    WR--------D--------------QARE----I---L-------------------------
Mariner2_MT_DNA    EALMASNTLHN--------------FMIQYKNTT---P-------------------------
BARI_DM-Tc1        -------------------------L------TLEFAQ-------------------------
FB4_DM-Tc1         --------------------------------PPKPCX-------------------------

cons

gi|152068700       ----------------KIIS-------------------------------------------
gi|300441029       ----------------AIIS-------------------------------------------
gi|571786598       ----------------KLID-------------------------------------------
gi|667676338       ----------------KLIE-------------------------------------------
gi|52548731        ----------------KLIK-------------------------------------------
Mariner-1_AP       ----------------NFVK-------------------------------------------
Mariner-2_AP       ----------------NFIS-------------------------------------------
Mariner-3_AP       ----------------NFIT-------------------------------------------
Mariner44_CB       -----VPRVFSSNFGRQTV--------------------------------------------
Submariner_Ac1     ----------------KAI--------------------------------------------
Submariner_Ac2     ----------------KSI--------------------------------------------
Cirt2_CA-Fot1      ----------------ELIDKLTKKIDFLELEN----------------TQ------------
Fot1_FO-Fot1       RLRAQLEAAQPK-KKRKIR------------------------------------------QD
Gizmo1_EI-Gizmo    ----------------DVVA-------------------------------------------
Gizmo2_EI-Gizmo    ----------------DVIA-------------------------------------------
MOGWAI1_EI-Mogw    ----------------RFFQEKRQEGELYRYNE----------------GKNNYQFEHKIIPD
MOGWAI2_EI-Mogw    ----------------SIXXSVFCENTLYQIE---------------------------ISPV
Mariner-1_NV-Po    ----------------FDQDDMTQEEDETNSDDNEPDVQIVGTKETTLTLAE-----------
Mariner2_MT_DNA    ----------------QLLDAIRKVRDELQTDL------------------------------
BARI_DM-Tc1        ----------------TLVRSIPKRLQAVIDAK------------------------------
FB4_DM-Tc1         ----------------DLV--------------------------------------------

cons

gi|152068700       ---------------------------------------------------------------
gi|300441029       ---------------------------------------------------------------
gi|571786598       ---------------------------------------------------------------
gi|667676338       ---------------------------------------------------------------
gi|52548731        ---------------------------------------------------------------
Mariner-1_AP       ---------------------------------------------------------------
Mariner-2_AP       ---------------------------------------------------------------
Mariner-3_AP       ---------------------------------------------------------------
Mariner44_CB       ---------------------------------------------------------------
Submariner_Ac1     ---------------------------------------------------------------
Submariner_Ac2     ---------------------------------------------------------------
Cirt2_CA-Fot1      ----------------LKTDVAHLQAELTSAKLEID---------------------------
Fot1_FO-Fot1       PNECF------------------------------------------ISLAQILAEANREPDQ
Gizmo1_EI-Gizmo    ---------------------------------------------------------------
Gizmo2_EI-Gizmo    ---------------------------------------------------------------
MOGWAI1_EI-Mogw    ISDTFAI----------------------------------QENNIMEEC----------DYY
MOGWAI2_EI-Mogw    LEDTNNIHELMTENNECSKNLVLTCSDKQDFHFHFEIDENTYSQNKIIDC----------DYE
Mariner-1_NV-Po    ----ATL----------------------------------HGE-------------------
Mariner2_MT_DNA    ---------------------------------------------------------------
BARI_DM-Tc1        ---------------------------------------------------------------
FB4_DM-Tc1         ---------------------------------------------------------------

cons

gi|152068700       ---------------------------------------------------------------
gi|300441029       ---------------------------------------------------------------
gi|571786598       ---------------------------------------------------------------
gi|667676338       ---------------------------------------------------------------
gi|52548731        ---------------------------------------------------------------
Mariner-1_AP       ---------------------------------------------------------------
Mariner-2_AP       ---------------------------------------------------------------
Mariner-3_AP       ---------------------------------------------------------------
Mariner44_CB       ---------------------------------------------------------------
Submariner_Ac1     ---------------------------------------------------------------
Submariner_Ac2     ---------------------------------------------------------------
Cirt2_CA-Fot1      N-------------YKEALKN------------------------------KKPPGRS-----
Fot1_FO-Fot1       RVIQSQKGDLD---CIVVDGKSSSE-----SEEDPA---PVR---------------------
Gizmo1_EI-Gizmo    ---------------------------------------------------------------
Gizmo2_EI-Gizmo    ---------------------------------------------------------------
MOGWAI1_EI-Mogw    NEEKLKSECSKSEYMFSQIST------------------------------IKCCDDIDKNTS
MOGWAI2_EI-Mogw    ANKTVDTLELS---DIHIINEQSTTYISQMEIDDPGNKINWKNVRQQHYSLNSMF------EY
Mariner-1_NV-Po    ---------------------------------------------------------------
Mariner2_MT_DNA    ---------------------------------------------------------------
BARI_DM-Tc1        ---------------------------------------------------------------
FB4_DM-Tc1         ---------------------------------------------------------------

cons

gi|152068700       ----------------------------------------------------------TVTEQ
gi|300441029       ----------------------------------------------------------KVFEQ
gi|571786598       ----------------------------------------------------------STITE
gi|667676338       ----------------------------------------------------------KVTLQ
gi|52548731        ----------------------------------------------------------KIWQQ
Mariner-1_AP       ----------------------------------------------------------HVIQI
Mariner-2_AP       ----------------------------------------------------------HTKKE
Mariner-3_AP       ----------------------------------------------------------HVTKE
Mariner44_CB       ---------------------------------------------------------SSHSKI
Submariner_Ac1     ---------------------------------------------------------AKVEKM
Submariner_Ac2     ---------------------------------------------------------LHAEKI
Cirt2_CA-Fot1      ---------------------------------------------------------SAIPMD
Fot1_FO-Fot1       -------------------------------------------------------RSTRVRRA
Gizmo1_EI-Gizmo    ----------------------------------------------------------SLERC
Gizmo2_EI-Gizmo    ----------------------------------------------------------SMEKC
MOGWAI1_EI-Mogw    PKLILPREIDE-STIDLIH-QKINVM-MNANVVLGLK--------------------------
MOGWAI2_EI-Mogw    LVFFNSRS-DIKELIEIIKTQRLNTLILNAFC-FWTKLRNLYEWGNPVFLFDWDNFWDGVINE
Mariner-1_NV-Po    ---------------------RLRQF-AQDKG-HE----------------------------
Mariner2_MT_DNA    ---------------------------------------------------------------
BARI_DM-Tc1        ---------------------------------------------------------------
FB4_DM-Tc1         ---------------------------------------------------------DFMPRG

cons

gi|152068700       EDIFW-KEDAE-T-------------------------------------------D------
gi|300441029       EERYW-AEDEK-LDE-------------------------------------------------
gi|571786598       EDSYW-KEDEK-IDN-NQGVD-------------------------------------------
gi|667676338       EEAFW-TED------------------------------------------------------
gi|52548731        EDLFW-TEDTE-ADE-RVTIE---------YKYTRLSENYI--DFEDE-------VY------
Mariner-1_AP       EDRFW-NVDMT-VDD-VMDDD---------NLHVMTITGDTSDSDDLG-CV-ALE--------
Mariner-2_AP       EDKFW-EIDFV-VDE-VLSAE---------LESVTLTIGDTSSDDSLG-TE-SDYFF------
Mariner-3_AP       EDKFW-QIDVL-SDE-LFDEQ---------EFHVLTITGDTSSDFDSD---------------
Mariner44_CB       GDRPS-RDDGRRRYDGL-------------------------RR-----GV-RLELRRR----
Submariner_Ac1     EDELF-VNDIY--DE--------------------------------------------I---
Submariner_Ac2     EEEMW-YD---------------------------------------------------D---
Cirt2_CA-Fot1      ENQGFKRAAPY-AKE-Y---RQNPPKKNKRKALTD---MTN-CT----NGS-NSYIRSL----
Fot1_FO-Fot1       TKMYI-RQDLS-SEE-------------------------------------------SD---
Gizmo1_EI-Gizmo    RNTVW-QKVFS-RDN-L----------------------------------------------
Gizmo2_EI-Gizmo    RNEVW-SLVYT-RSD-L----------------------------------------------
MOGWAI1_EI-Mogw    -------------------------------------------------------FN------
MOGWAI2_EI-Mogw    ERIRG-QMDEI-IDE-D---V----------------------------ILIPVYYAEHFMLI
Mariner-1_NV-Po    --EL--SLLFTRAND-I---------------LYS---LQLAGQKK-QTTV-DSFL-------
Mariner2_MT_DNA    -----------------------------------------NFKGK-QTTI-ESYFNKV----
BARI_DM-Tc1        ------------------------------------------------GGV-TK----Y----
FB4_DM-Tc1         CKAVL-ANKGY-PAK-------------------------------------------Y----

cons

gi|152068700       ------
gi|300441029       ------
gi|571786598       ------
gi|667676338       ------
gi|52548731        ------
Mariner-1_AP       ------
Mariner-2_AP       ------
Mariner-3_AP       ------
Mariner44_CB       ------
Submariner_Ac1     ------
Submariner_Ac2     ------
Cirt2_CA-Fot1      ------
Fot1_FO-Fot1       ------
Gizmo1_EI-Gizmo    ------
Gizmo2_EI-Gizmo    ------
MOGWAI1_EI-Mogw    ------
MOGWAI2_EI-Mogw    GVFLKH
Mariner-1_NV-Po    ------
Mariner2_MT_DNA    ------
BARI_DM-Tc1        ------
FB4_DM-Tc1         ------

cons
```
