# Supplementary material for: DNA transposons have colonized the genome of the giant virus Pandoravirus salinus
Source: BMC Biol. 2015 Jun 12;13:38. doi: 10.1186/s12915-015-0145-1 (PMC4495683; doi:10.1186/s12915-015-0145-1)
Supplement: Additional file 7: Figure S7. — Pairwise alignment of the miniature inverted-repeat transposable element (MITE) in the A. castellanii genome and the MITE in P. salinus (Submariner_Ps1) that has the highest sequence similarity (out of the 30 MITE copies) with the MITE in A. castellanii. [file 12915_2015_145_MOESM7_ESM.pdf]

```
                                               1
MITEinA.castellanii(AEYA01002349.1:6514-6782)  CAGTCTCCTA ACTCTCAAAG GGCCAAAA-- -AAGTCAAAA
MITEinP.salinus(KC977571.1:17709-17950)        CAGTCCCCGA ACCCTCAAAG GGGGCAAAAT AAAGTCATAA

                                              41
MITEinA.castellanii(AEYA01002349.1:6514-6782)  CTGGTCAATG CGCAACCTCC GGT--ACCGG TCGCACGTTG
MITEinP.salinus(KC977571.1:17709-17950)        AAAGTCAAAG GGACGTCCCA AAAATGTCTA CAGCCTGTTG

                                              81
MITEinA.castellanii(AEYA01002349.1:6514-6782)  TTGTGCCATA CGAAACCTG- ---------- --TTTCAAAC
MITEinP.salinus(KC977571.1:17709-17950)        TTTCGTCTGC TTAAAAATTG TAGGCATATG GGGGTGAGAC

                                             121
MITEinA.castellanii(AEYA01002349.1:6514-6782)  CTGTCTGTCC GCAGACGCAC CAGGTGAGCC AAGAAGGCAA
MITEinP.salinus(KC977571.1:17709-17950)        ATGTCTGATG TCGGCACTTT CG------CG CACTCCCAAG

                                             161
MITEinA.castellanii(AEYA01002349.1:6514-6782)  CGACCAAAAA AGAGCTCTTT TTAGCTATTT TTTGGCCCTA
MITEinP.salinus(KC977571.1:17709-17950)        CGG--ACAAA ACAACAGGCT GTAGACATTT TGGGACGCCC

                                             201
MITEinA.castellanii(AEYA01002349.1:6514-6782)  CTCCACACGT TGAAAAAAAG CAATTTTAAT AGGCGAAAAA
MITEinP.salinus(KC977571.1:17709-17950)        CTTT------ ---------- ---------- ----------

                                             241
MITEinA.castellanii(AEYA01002349.1:6514-6782)  AGCCAATGAA CCGGCGACAT TCGACCCCTT TGAGAGTTCA
MITEinP.salinus(KC977571.1:17709-17950)        -GACTTTTTA TGACTTTATT TTGCCCCCTT TGAGAGTTCG

                                             281
MITEinA.castellanii(AEYA01002349.1:6514-6782)  GAGACTG
MITEinP.salinus(KC977571.1:17709-17950)        GGGACTG
```
